# Cost-effectiveness of Increasing Buprenorphine Treatment Initiation, Duration, and Capacity Among Individuals Who Use Opioids

**DOI:** 10.1001/jamahealthforum.2023.1080

**Published:** 2023-05-19

**Authors:** Anneke L. Claypool, Catherine DiGennaro, W. Alton Russell, Melike F. Yildirim, Alan F. Zhang, Zuri Reid, Erin J. Stringfellow, Benjamin Bearnot, Bruce R. Schackman, Keith Humphreys, Mohammad S. Jalali

**Affiliations:** 1 Massachusetts General Hospital Institute for Technology Assessment, Harvard Medical School, Boston; 2 School of Population and Global Health, McGill University, Montreal, Quebec, Canada; 3 Division of General Internal Medicine, Massachusetts General Hospital, Boston; 4 Department of Population Health Sciences, Weill Cornell Medical College, New York, New York; 5 Veterans Affairs and Stanford University Medical Centers, Palo Alto, California; 6 MIT Sloan School of Management, Cambridge, Massachusetts

## Abstract

**Importance:**

Buprenorphine is an effective and cost-effective medication to treat opioid use disorder (OUD), but is not readily available to many people with OUD in the US. The current cost-effectiveness literature does not consider interventions that concurrently increase buprenorphine initiation, duration, and capacity.

**Objective:**

To conduct a cost-effectiveness analysis and compare interventions associated with increased buprenorphine treatment initiation, duration, and capacity.

**Design and Setting:**

This study modeled the effects of 5 interventions individually and in combination using SOURCE, a recent system dynamics model of prescription opioid and illicit opioid use, treatment, and remission, calibrated to US data from 1999 to 2020. The analysis was run during a 12-year time horizon from 2021 to 2032, with lifetime follow-up. A probabilistic sensitivity analysis on intervention effectiveness and costs was conducted. Analyses were performed from April 2021 through March 2023. Modeled participants included people with opioid misuse and OUD in the US.

**Interventions:**

Interventions included emergency department buprenorphine initiation, contingency management, psychotherapy, telehealth, and expansion of hub-and-spoke narcotic treatment programs, individually and in combination.

**Main Outcomes and Measures:**

Total national opioid overdose deaths, quality-adjusted life years (QALYs) gained, and costs from the societal and health care perspective.

**Results:**

Projections showed that contingency management expansion would avert 3530 opioid overdose deaths over 12 years, more than any other single-intervention strategy. Interventions that increased buprenorphine treatment duration initially were associated with an increased number of opioid overdose deaths in the absence of expanded treatment capacity. With an incremental cost- effectiveness ratio of $19 381 per QALY gained (2021 USD), the strategy that expanded contingency management, hub-and-spoke training, emergency department initiation, and telehealth was the preferred strategy for any willingness-to-pay threshold from $20 000 to $200 000/QALY gained, as it was associated with increased treatment duration and capacity simultaneously.

**Conclusion and Relevance:**

This modeling analysis simulated the effects of implementing several intervention strategies across the buprenorphine cascade of care and found that strategies that were concurrently associated with increased buprenorphine treatment initiation, duration, and capacity were cost-effective.

## Key Points

**Question:**

Are interventions to increase buprenorphine treatment initiation, duration, and capacity for treating opioid use disorder cost-effective?

**Findings:**

In this quality and economic evaluation of modeled interventions to increase buprenorphine treatment initiation, duration, and capacity, we projected health outcomes and costs of 5 interventions and their combinations. This study found that a portfolio of interventions, including contingency management, hub-and-spoke clinician training, initiation of buprenorphine treatment in the emergency department, and telehealth, was the preferred strategy for a range of generally accepted willingness-to-pay thresholds.

**Meaning:**

The findings of this study suggest that interventions that increase buprenorphine treatment duration concurrently with treatment capacity are the most cost-effective and have the largest association with reducing overdose deaths.

## Teaser

This economic evaluation study includes a cost-effectiveness analysis and compares interventions associated with increased buprenorphine treatment initiation, duration, and capacity.

## Introduction

The opioid crisis remains a major public health challenge in the US. Between 1999 and 2020, overdose deaths involving opioids surpassed 564 000,^1^ with a record 75 673 opioid overdose deaths (OODs) in 2021.^2^ As of 2019, about 1.4 million people had opioid use disorder (OUD),^3^ and only an estimated 34.5% received OUD treatment that year.^4^ One effective way to reduce fatal opioid overdoses is to increase the uptake and duration of medication treatment for OUD (MOUD), including buprenorphine.^5^

Buprenorphine has the potential to be expanded, as it can be administered outside of specialty outpatient facilities and is cost-effective.^6^ Several local-level and state-level interventions have been associated with improved buprenorphine initiation (ie, the number initiating treatment), duration (ie, average length of treatment), and capacity (ie, the number of patients that available prescribers can treat). For example, making buprenorphine available in the emergency department is associated with increased initiation^7,8^; contingency management, psychotherapy, and telehealth are associated with increased duration^9^; and hub-and-spoke networks are associated with increased capacity.^10^

Prior analyses estimated that buprenorphine treatment is effective, so expanding its use could be associated with large health benefits and reduced mortality.^9,11,12,13,14^ However, studies have not considered how increases in buprenorphine use may affect capacity and how limited capacity may affect buprenorphine effectiveness and cost-effectiveness. Recent regulatory changes may expand capacity by increasing the number of buprenorphine prescribers. Until 2021, physicians and other eligible health care clinicians in the US were required to complete at least 8 hours of training to obtain a waiver allowing them to prescribe buprenorphine to 30 patients or fewer.^15^ This requirement was lifted in 2021 to allow prescribing to up to 30 patients per clinician without a waiver or additional training by notifying the federal government.^16^ The waiver requirement was lifted in December 2022, allowing clinicians to prescribe buprenorphine with a standard US Drug Enforcement Administration prescribing registration number.^17^ Recent increases in the availability of clinician training and ongoing support for buprenorphine prescribers may also increase treatment capacity.^10^ The objective of this study was to leverage modeling to analyze the cost-effectiveness of interventions and combinations of interventions to expand buprenorphine treatment in the US while considering (1) the tradeoffs of increasing buprenorphine duration, initiation, and capacity and (2) the interaction between the demand for treatment and treatment capacity.

## Methods

### Model and Interventions

We used the SOURCE model^18,19^ to estimate the potential public health effects and assess cost-effectiveness of 5 interventions, and their combinations, aimed at increasing OUD treatment with buprenorphine. SOURCE is a system dynamics model that tracks opioid misuse, including OUDs involving prescription opioids and heroin. It models treatment with MOUD, remission, relapse, nonfatal opioid overdose, and OOD. eMethods 1 and eFigure 1 in Supplement 1 present more information about SOURCE. The Mass General Brigham institutional review board exempted the study from review and waived informed consent because the data were not obtained through participant interaction.

We compared 5 interventions: contingency management (CM), psychotherapy, a hub-and-spoke training and support model, emergency department (ED)–initiated buprenorphine, and telehealth for buprenorphine prescribing. Emergency department–initiated buprenorphine entails initiating treatment with MOUD while someone with OUD is in the ED and uses bridge clinics to link them to continued buprenorphine care postdischarge. Contingency management gives patients a voucher, prize, or cash-based incentive for continuing treatment or achieving a negative toxicology screen result.^20^ Psychotherapy is often paired with MOUD to offer mental health support and may be used to treat underlying mental illness or trauma, while some clinicians require psychotherapy to receive MOUD,^21^ making it a potential barrier that can decrease duration. Telehealth allows patients taking buprenorphine to meet with health care clinicians through video or phone visits. Policy changes enacted during the COVID-19 pandemic allow greater freedom for clinicians to initiate and continue buprenorphine treatment via telehealth.^22^ Finally, a hub-and-spoke model increases training and support for clinicians, which uses existing opioid treatment programs that are authorized to prescribe buprenorphine as hubs, while community primary care practices function as the spokes of the program. Patients are referred to the spokes once they are stabilized with treatment with MOUD, while the hubs provide mentorship and training in buprenorphine prescribing.^10,23,24^

### Literature Review, Effects, and Costs of Interventions

The 5 interventions are associated with increases in the initiation, duration, or capacity of buprenorphine treatment. Emergency department initiation provides support for linking to buprenorphine care, which is followed by increased buprenorphine initiation.^7,8^ Contingency management encourages patients to continue to receive treatment, which is associated with increased average treatment duration.^9^ Psychotherapy can encourage patients to continue to receive treatment, but may also serve as a barrier when it is a requirement before receiving buprenorphine, and has an average positive association with increasing duration, but with uncertainty that includes the possibility of shortening duration.^9,21^ Telehealth is also associated with increased duration by reducing barriers caused by distance and transportation to prescriber offices, allowing people to continue to receive treatment longer.^25^ Hub-and-spoke models include training and ongoing mentorship to clinicians, and research shows they are associated with an increased total number of prescribers and number of patients per prescriber.^10,23,24,25^

We reviewed the literature for estimates of the association of each intervention with duration, capacity, and initiation and the cost of each intervention (eMethods 2 and eTables 1-6 in Supplement 1). Based on previous analyses, the model assumed that lifting the X waiver was not associated with a change in initiation, capacity, or duration.^26,27,28^ We included the type of effect for each intervention (duration, capacity, and initiation) based on measured effects in the literature and excluded effects that we could not quantify. The cost of hub-and-spoke was estimated per clinician based on program-funding data and the total number of clinicians included in the program (eMethods 3 in Supplement 1).^23^ Telehealth is estimated to reduce costs compared with in-person visits.^29^ These cost reductions are for clinicians (eg, by better time allocation) and patients (eg, by removing the burden of travel, which patients often report as a barrier to MOUD treatment).^30^ Studies have found telehealth to have lower costs than in-person appointments for various appointment types (eTable 7 in Supplement 1). We made a conservative assumption of no cost savings for telehealth compared with in-person visits. Costs of the other interventions were based on the literature (Table 1^7,9,10,13,31,32,33,34,35,36,37,38,39,40,41,42,43,44,45,46,47,48,49,50^; eMethods 3 in Supplement 1) and adjusted for inflation to 2021 USD.

**Table 1.** Parameter Values and Sources

Parameter	Value (uncertainty range)	Source
**Intervention effectiveness** ^a^		
Initiation		
Emergency department buprenorphine initiation: relative rate of buprenorphine treatment initiation	1.556 (1.164-2.108)	^7,31,32,33^
Duration		
Contingency management: hazard rate ratio of buprenorphine discontinuation	0.594 (0.437-0.787)	^9^
Psychotherapy: hazard rate ratio of buprenorphine discontinuation	0.986 (0.772-1.240)	^9^
Telehealth: hazard rate ratio of buprenorphine discontinuation	0.69 (0.60-0.78)	^50^
Capacity		
Hub-and-spoke: percentage increase in the total number of clinicians^b^	64%	^10^
Hub-and-spoke: percentage increase in the number of patients per clinician^b^	50%	^10^
Intervention coverage (percentage of population covered by intervention)	10% (5%-20%)	Assumed
**QALYs for each health state^c^**		
Nondisordered heroin use	0.574 (0.538-0.611)	^34^
Rx misuse, no heroin use in the past year	0.694 (0.660-0.727)	^34^
Rx OUD, no heroin use in the past year, no MOUD	0.626 (0.591-0.661)	^34^
Rx OUD with heroin use in the past year, no MOUD	0.569 (NA)	Calculated
HUD no MOUD	0.512 (0.475-0.549)	^34^
Rx OUD or HUD in MOUD treatment	0.7660 (0.7395-0.7925)	^34^
Rx OUD or HUD in remission	0.807 (0.78-0.834)	^34^
Average additional QALYs for lifetime follow-up	22.64	^35,36,37^
**Costs (2021 USD)^d^**		
Cost of hub-and-spoke training program per clinician in the network^e^	34 558	^23^
Contingency management per patient year^e^	3578 (2684-4473)	^9^
Psychotherapy per patient year^e^	4540 (3405-5675)	^9,38^
Emergency department–initiated buprenorphine per patient^e^	558 (418-697)	^8,39^
Telehealth per patient	0	Assumed^f^
Annual background health care costs	6063	^40^
Annual excess cost of health care utilization with OUD: out of treatment	7598	^41^
Annual excess cost of health care utilization with OUD: in treatment	6078	^9,41,42^
Annual methadone treatment^e^	7294 (5471-9118)	^9,38^
Annual buprenorphine treatment^e^	6650 (4988-8313)	^9,38^
Annual extended-release naltrexone treatment^e^	15 674 (11 756-19 593)	^9,38^
Nonfatal overdose emergency and inpatient	4377 (3342-4793)	^43,44,45,46,47^
Fatal overdose emergency and inpatient	3568 (2758-4562)	^43,44,45,46,47^
Remaining lifetime productivity for average age 35-49 y, included per opioid overdose death	1 517 261	^36,37,48,49^
Remaining lifetime consumption for average age 35-49 y, included per opioid overdose death	745 408	^36,37,49^
**Average annual criminal justice cost by health state (2021 USD)** ^**d**^ **^,^ ^g^**		
Annual criminal justice cost: OUD, not in treatment	2616 (2250-2983)	^13^
Annual criminal justice cost: OUD, in treatment	566 (473-649)	^13^
Annual criminal justice cost: OUD, in remission	343 (295-391)	^13^
Annual criminal justice cost: HUD, not in treatment	4210 (3871-4549)	^13^
Annual criminal justice cost: HUD, in treatment	1067 (1150-1497)	^13^
Annual criminal justice cost: HUD in remission	551 (507-596)	^13^

Abbreviations: HUD, opioid use disorder involving heroin; MOUD, medication for opioid use disorder; NA, not applicable; OUD, opioid use disorder; Rx OUD, opioid use disorder involving prescription opioids; QALY, quality-adjusted life-year.

^a^ Further details on intervention effectiveness as applied in SOURCE are included in eTable 8 in Supplement 1. All other parameters were previously published.^19^

^b^ Hub-and-spoke capacity effects and uncertainty ranges were converted for input into the model (eTable 8 in Supplement 1).

^c^ Detailed QALYs per health state are included in eTable 14 in Supplement 1.

^d^ Adjusted for inflation to reflect 2021 USD using the Consumer Price Index.

^e^ Uncertainty range based on a 25% range more or less than the base case value.

^f^ Telehealth cost is estimated to be 0 based on the conservative assumption that it will not save costs (eMethods 3 and eTable 7 in Supplement 1).

^g^ Criminal justice costs include the price of incarceration.

### Projecting the Effects of Interventions

Using SOURCE, we projected the status quo of current buprenorphine prescribing and the associated health outcomes using the same parameters of previous analyses.^18,19^ We then analyzed the status quo, each intervention alone, and all combinations of interventions, a total of 32 strategies, based on the estimated association of these strategies with buprenorphine treatment initiation, capacity, and duration (eTable 8 in Supplement 1).

To simulate a proportion of the population who would feasibly receive a given intervention on a national scale, we assumed a coverage level of 10% of the total number of patients and prescribers affected by each strategy. The assumption of 10% intervention coverage was based on expert opinion and is subject to the scenario analyses. We assumed a 3-year linear ramp-up period for each strategy’s coverage starting in 2022 and reaching full coverage by 2024. We also assumed that no strategy was associated with buprenorphine diversion for illicit use.

### Health States and Outcomes

Total quality-adjusted life years (QALYs) reflect the disutility associated with OUD. We determined the quality-of-life effects of treatment from a literature search^34^ (Table 1) and applied QALYs to the number of people in each health state in SOURCE as stratified by type of opioid use, treatment status, and remission status. We projected the effectiveness of each intervention during a 12-year horizon (2021-2032) with lifetime follow-up, which includes average lifetime QALYs and costs expected for each person remaining in the model after 2032 as based on the age-weighted quality-adjusted life expectancy (Table 1; eMethods 4 in Supplement 1). We estimated total nonfatal opioid overdoses, OODs, and QALYs and compared these health outcomes with the status quo of no additional interventions.^18,19^

### Cost-effectiveness Analysis

In the primary cost-effectiveness analysis, we took a societal perspective. Total costs included background and excess health care costs by OUD and treatment status, treatment costs, criminal justice costs, and intervention costs based on the number of people in each health state (Table 1; eMethods 5 and 6 in Supplement 1). For each overdose death, the age-weighted lost net productivity (ie, the expected productivity for each person minus the expected consumption during the remainder of their lifetime) was included.^51^

For each strategy, we calculated average costs and QALYs for each health and treatment state and costs for each intervention (Table 1), applying an annual discount rate of 3% for all costs and QALYs.^52^ We calculated the incremental cost-effectiveness ratios (ICERs) for each strategy by calculating the incremental costs divided by incremental QALYs compared with the strategy with the next lowest cost. Strategies were considered dominated if they were more expensive and less effective than alternative strategies. We compared the resulting ICERs with a range of willingness-to-pay thresholds up to $200 000/QALY gained, for which strategies are generally considered to be cost-effective in the US.^53,54,55^ We followed the Consolidated Health Economic Evaluation Reporting Standards (CHEERS) reporting guideline.^56^

### Sensitivity Analysis

We conducted a probabilistic sensitivity analysis to vary the effectiveness and costs of each of the interventions and the costs of MOUD (Table 1) for 1000 runs using uniform distributions. Costs were varied 25% from the base case. To compare the interventions, the incremental net monetary benefit was calculated with a willingness to pay of $100 000/QALY gained. Interventions with positive net monetary benefits were considered cost-effective compared with the status quo, and those with the highest incremental net monetary benefit were considered the preferred strategy.

### Health Care Perspective and Scenario Analysis

We compared the base case results from a societal perspective with those from the health care perspective, which excludes criminal justice, productivity, and consumption costs. We also compared the results of the cost-effectiveness assessment by estimating the effect of varying intervention coverage from 10% to 5% and 20% for each intervention. eMethods 7 in Supplement 1 presents the estimation process.

### Study Timeline and Data Use

The analysis was conducted from March 2021 through March 2023. Data inputs were based on published literature or on the previously calibrated and validated SOURCE model. Vensim DSS (Ventana Systems) was used for the simulation analysis and R (R Foundation) was used to calculate the cost-effectiveness results.

## Results

### Health Outcomes

In the status quo, SOURCE projected 7 593 200 nonfatal and 668 424 fatal opioid overdoses from 2021 to 2032. We estimated that buprenorphine treatment is currently capacity constrained (patient demand exceeds available care). Within the status quo, we projected that, due to a declining trend in the total number of people with OUD,^18,19^ buprenorphine treatment demand will decrease^28^ and no longer be capacity constrained as of 2024 (eFigure 2 in Supplement 1).

**Figure 1.**
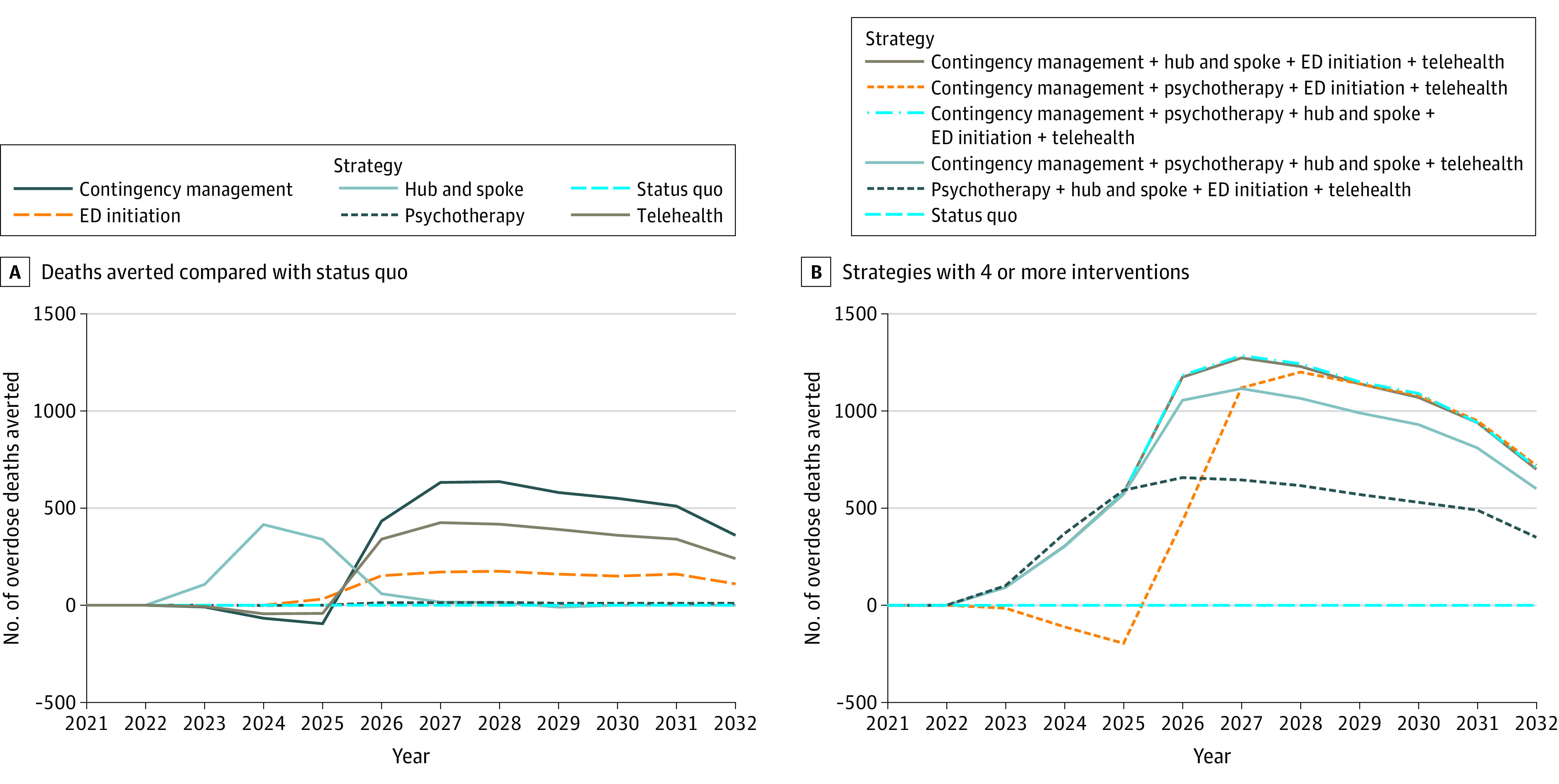
Overdose Deaths Averted Compared With the Status Quo, 2021-2032 Line graphs that show the overdose deaths averted, calculated by the number of overdose deaths in the status quo subtracted by the number of overdoses for each intervention, each year from 2021 to 2032. A, Single-intervention strategies. B, Portfolio strategies with 4 or more interventions. ED indicates emergency department initiation.

Figure 1 presents the projected trends of overdose deaths compared with the status quo from 2021, 1 year before the interventions, to 2032. Starting in 2026, the expansion of CM services for buprenorphine averted the most annual overdose deaths compared with any other single-intervention strategies, after increasing deaths initially due to increased demand, which constrained the limited available capacity (Figure 1A). Expansion of the hub-and-spoke model initially predicted an increase in overdose deaths averted, which declined to 0 as capacity became less constrained. Contingency management, which was operationalized with an increase in the average treatment duration of 7% with a coverage level of 10% of patients with buprenorphine, was associated with more deaths than the status quo from 2021 to 2025, when buprenorphine treatment capacity was limited, because patients received treatment longer, decreasing the capacity available for new patients to initiate treatment with buprenorphine. Implementing interventions associated with increased capacity alongside interventions associated with increased treatment duration was followed by more deaths averted and no initial increase in deaths (Figure 1B).

From 2021 to 2032, CM had the largest health benefit of all the single-intervention strategies, averting 3530 fatal overdoses (Table 1) and gaining 182 127 QALYs (Table 2) compared with the status quo. The least beneficial single-intervention strategy was the expansion of psychotherapy, which was estimated to avert 80 overdose deaths (Table 1) and gain 4238 QALYs. The combination of all 5 interventions averted 8570 overdose deaths (Table 1) and gained 520 557 QALYs, the most of any strategy combination (Table 2).

**Table 2.** Cumulative Expected Health Outcomes and Comparison With the Status Quo From 2021 to 2032

Strategy	Total nonfatal opioid overdoses, 2021-2032	Total nonfatal opioid overdoses averted^a^	Total fatal opioid overdoses, 2021-2032	Total opioid overdose deaths averted^a^
SQ	7 593 200	0	668 424	0
**Single-intervention strategies**				
CM	7 562 800	30 400	664 894	3530
P	7 592 500	700	668 344	80
HS	7 584 200	9000	667 484	940
ED	7 583 200	10 000	667 314	1110
TH	7 572 300	20 900	666 004	2420
**Strategies with multiple interventions**				
CM+P	7 562 200	31 000	664 834	3590
CM+HS	7 548 400	44 800	663 374	5050
P+HS	7 583 500	9700	667 394	1030
CM+P+HS	7 547 700	45 500	663 294	5130
CM+ED	7 554 000	39 200	663 924	4500
HS+ED	7 573 600	19 600	666 294	2130
P+ED	7 582 600	10 600	667 234	1190
CM+P+ED	7 553 500	39 700	663 864	4560
CM+HS+ED	7 538 300	54 900	662 254	6170
P+HS+ED	7 572 800	20 400	666 214	2210
CM+P+HS+ED	7 537 600	55 600	662 174	6250
CM+TH	7 546 800	46 400	663 034	5390
P+TH	7 571 700	21 500	665 934	2490
HS+TH	7 560 200	33 000	664 734	3690
ED+TH	7 563 100	30 100	664 984	3440
CM+P+TH	7 546 400	46 800	662 984	5440
CM+HS+TH	7 527 400	65 800	660 964	7460
P+HS+TH	7 559 500	33 700	664 644	3780
CM+ED+TH	7 538 900	54 300	662 164	6260
HS+ED+TH	7 549 900	43 300	663 584	4840
P+ED+TH	7 562 600	30 600	664 914	3510
CM+P+ED+TH	7 538 500	54 700	662 104	6320
CM+HS+ED+TH	7 517 900	75 300	659 924	8500
P+HS+ED+TH	7 549 100	44 100	663 504	4920
CM+P+HS+TH	7 526 800	66 400	660 894	7530
CM+P+HS+ED+TH	7 517 300	75 900	659 854	8570

Abbreviations: CM, contingency management; ED, emergency department initiation; HS, hub and spoke; P, psychotherapy; SQ, status quo; TH, telehealth; QALYs, quality-adjusted life-years.

^a^ Nonfatal and fatal overdoses averted compared with the status quo of no intervention.

Important effects from the combinations of interventions were captured by the model (Table 2). The strategy of telehealth and hub-and-spoke averted 3690 fatal overdoses, more than adding the respective fatal overdoses averted of telehealth (2420) and hub-and-spoke (940), showing a synergy when increasing duration and capacity. The strategy of CM and telehealth averted fewer fatal overdoses (5390) than the sum of overdose deaths averted by CM (3530) and telehealth (2420), suggesting diminishing returns when implementing interventions that increase duration without also increasing capacity.

### Expected Costs and Cost-effectiveness

Table 3 shows cost-effectiveness results for all strategies. The portfolio of psychotherapy and hub-and-spoke was the strategy with the highest costs, and the portfolio of all interventions had the most QALYs gained. For any willingness-to-pay threshold between $20 000 to $200 000/QALY gained, the strategy with CM, hub-and-spoke, ED initiation, and telehealth was the preferred strategy with an ICER of $19 381/QALY gained (Table 3).

**Table 3.** Expected Intervention QALYs, Costs, and ICER From 2021 to 2032

Strategy	QALYs gained compared with SQ	Total QALYs	Incremental cost compared with SQ (2021 USD)	Total cost (2021 USD)	ICER (2021 USD per QALY gained)^a^
CM+ED+TH	341 400	236 273 859	−2 782 819 715	1 568 705 665 750	ND
ED+TH	209 558	236 142 017	−2 435 967 847	1 569 052 517 617	D
CM+TH	269 948	236 202 407	−2 144 103 592	1 569 344 381 873	D
TH	126 866	236 059 326	−1 695 331 982	1 569 793 153 483	D
CM+ED	260 774	236 193 233	−1 531 093 743	1 569 957 391 722	D
CM	182 127	236 114 587	−825 495 129	1 570 662 990 336	D
ED	89 696	236 022 155	−807 012 013	1 570 681 473 452	D
CM+P+ED+TH	343 508	236 275 967	−722 016 921	1 570 766 468 544	Ex.D
P+ED+TH	212 716	236 145 176	−395 941 485	1 571 092 543 980	D
CM+P+TH	272 254	236 204 714	−80 729 974	1 571 407 755 491	D
SQ	0	235 932 459	0	1 571 488 485 465	D
P+TH	130 344	236 062 803	340 749 222	1 571 829 234 687	D
CM+P+ED	263 770	236 196 230	520 045 349	1 572 008 530 814	D
CM+HS+ED+TH	517 045	236 449 504	621 365 463	1 572 109 850 928	19 381
P+ED	93 729	236 026 188	1 214 388 568	1 572 702 874 033	D
CM+P	185 185	236 117 644	1 222 085 215	1 572 710 570 680	D
CM+HS+TH	431 933	236 364 392	1 381 620 059	1 572 870 105 524	D
HS+ED+TH	321 292	236 253 752	1 572 431 231	1 573 060 916 695	D
P	4238	235 936 697	2 017 612 041	1 573 506 097 505	D
CM+HS+ED	393 612	236 326 072	2 282 961 431	1 573 771 446 896	D
HS+TH	228 526	236 160 985	2 409 725 222	1 573 898 210 687	D
CM+P+HS+ED+TH	520 557	236 453 017	2 682 034 110	1 574 170 519 574	586 639
CM+HS	302 647	236 235 107	3 098 572 950	1 574 587 058 415	D
CM+P+HS+TH	435 857	236 368 316	3 438 915 984	1 574 927 401 449	D
HS+ED	172 391	236 104 850	3 474 166 143	1 574 962 651 608	D
P+HS+ED+TH	325 857	236 258 317	3 609 005 380	1 575 097 490 845	D
CM+P+HS+ED	397 676	236 330 136	4 326 163 270	1 575 814 648 735	D
HS	76 461	236 008 921	4 337 293 541	1 575 825 779 006	D
P+HS+TH	233 116	236 165 576	4 436 932 348	1 575 925 417 813	D
CM+P+HS	306 988	236 239 448	5 138 220 514	1 576 626 705 979	D
P+HS+ED	177 055	236 109 514	5 498 163 482	1 576 986 648 947	D
P+HS	81 505	236 013 964	6 351 765 629	1 577 840 251 094	D

Abbreviations: CM, contingency management; D, dominated; ED, emergency department initiation; Ex.D, extended dominance; HS, hub and spoke; ICER, incremental cost-effectiveness ratio; ND, not dominated; P, psychotherapy; SQ, status quo; TH, telehealth.

^a^ Strategies that are D are more costly and less effective than ND strategies. Strategies that are dominated by extension are more costly and less effective than the linear scale-up ND strategies.

**Figure 2.**
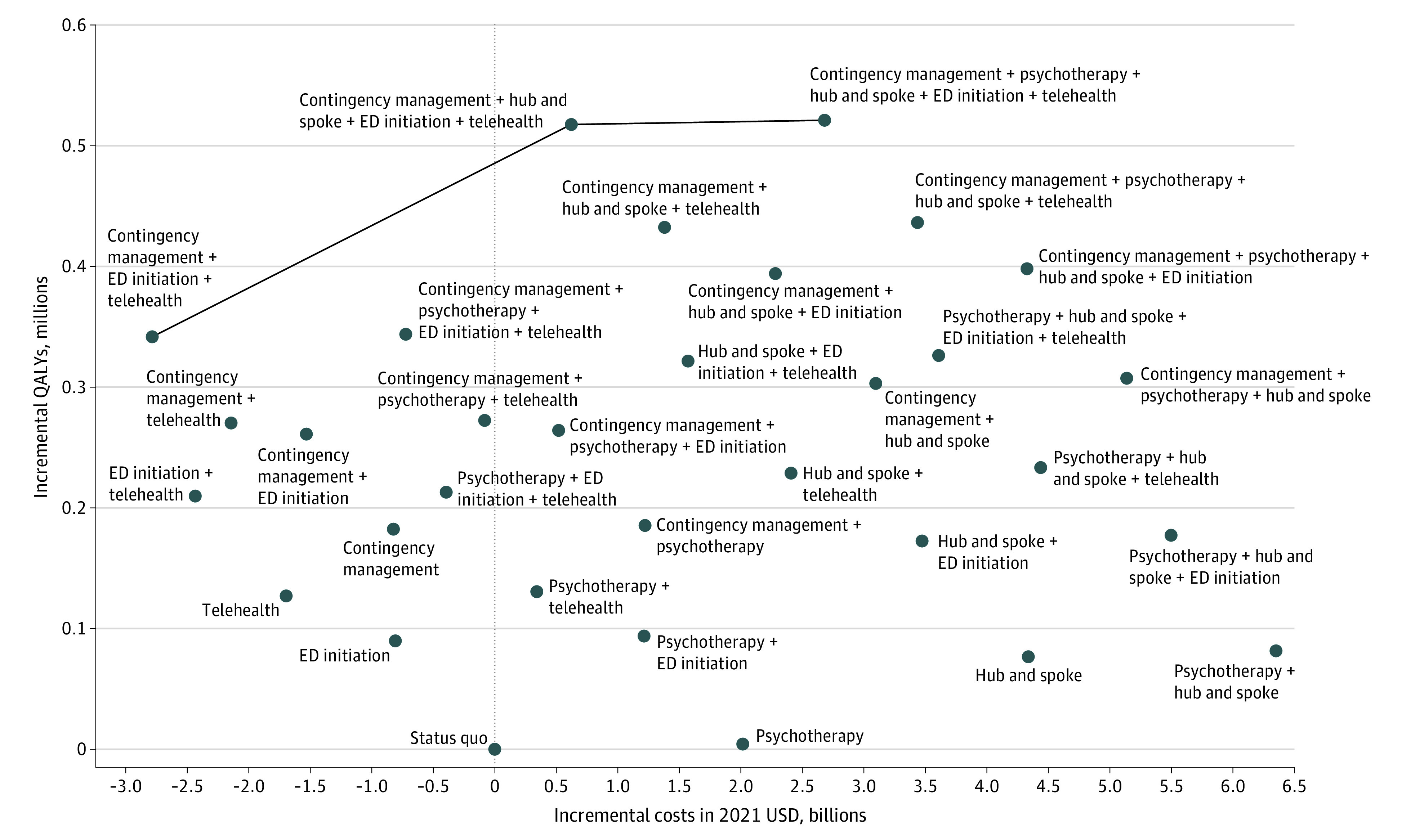
Cost-effectiveness Plane The x-axis shows incremental costs compared with the status quo, and the y-axis shows quality-adjusted life-years (QALYs) gained compared with the status quo. Strategies along the line are on the cost-effectiveness frontier and are more efficient than other strategies, as they cost less while having a higher health benefit than alternative strategies. ED indicates emergency department initiation.

Emergency department initiation + telehealth + CM, CM + hub-and-spoke + ED initiation + telehealth, and the strategy with all 5 interventions were along the cost-effectiveness frontier (Figure 2). Ten strategies were cost-saving compared with the status quo, as they had greater health benefits with lower total costs than the status quo.

### Sensitivity Analysis

Cost-effectiveness results were robust across uncertainty ranges. When considering a range of effectiveness in 1000 simulation runs, 10 strategies were cost-effective compared with the status quo in 100% of runs with a willingness-to-pay threshold of $100 000 per QALY gained (eTable 9 in Supplement 1). The combination of CM, hub-and-spoke, ED initiation, and telehealth was the preferred intervention in 66% of probabilistic sensitivity analysis runs and the combination of all interventions was preferred in 34% of runs. Apart from psychotherapy alone and the combination of psychotherapy with hub-and-spoke, all other strategies were cost-effective compared with the status quo for any willingness-to-pay threshold of less than $100 000/QALY gained for at least 70% of runs. The combination of CM, ED initiation, and telehealth was preferred most often for a willingness-to-pay threshold less than $16 500, and the combination of CM, hub-and-spoke, ED initiation, and telehealth was preferred most often greater than that threshold (eFigure 3 in Supplement 1).

### Health Care Perspective and Scenario Analysis

When we considered only the health care perspective, the strategy with CM, hub-and-spoke, ED initiation, and telehealth remained the preferred strategy for any willingness-to-pay threshold between $30 000 to $200 000/QALY gained, with an ICER of $29 514/QALY gained (eTable 10 in Supplement 1). Emergency department initiation remained cost-saving compared with the status quo in the health care perspective.

When comparing the results of an expansion of 10% coverage to 5% and 20% for all interventions, the number of overdose deaths averted increased with increasing intervention coverage, but it was less as the rate of coverage level increased (eTable 11 in Supplement 1). For example, the strategy with all interventions averted 4360 overdose deaths with 5% coverage and 8570 overdose deaths with 10% coverage and averted 16 620 overdose deaths for 20% coverage (eTable 11 in Supplement 1). The strategy with CM, hub-and-spoke, ED initiation, and telehealth remained the preferred strategy, with an ICER of $27 776/QALY gained for a coverage level of 5% and $11 531/QALY gained for a coverage level of 20% (eTables 12 and 13 in Supplement 1).

## Discussion

In this economic evaluation study that projected health outcomes and cost-effectiveness from 2021 to 2032, we found that a combination of strategies, including CM, hub-and-spoke clinician training, ED buprenorphine initiation, and telehealth was the preferred strategy at a generally accepted threshold and was likely to be cost-saving compared with the status quo. In sensitivity analyses, several combinations of interventions associated with increased buprenorphine treatment duration, capacity, and treatment initiation were considered cost-effective vs a plausible range of parameter values. Many strategies were cost-effective or cost-saving compared with the status quo, illustrating that various policy options associated with increased buprenorphine treatment and improved health outcomes are preferable compared with current treatment levels.

Although CM and telehealth were projected to be effective in increasing treatment duration, this strategy could be associated with an initial increase in overdose deaths due to treatment capacity constraints. However, when strategies combined interventions that were associated with increased treatment duration and capacity, there was a decrease in overdose deaths, and the benefits were greater than the sum of the 2 separate interventions, indicating the importance of increasing duration and capacity simultaneously. On the other hand, only increasing capacity through the hub-and-spoke training had a limited effect and only averted overdose deaths when buprenorphine treatment capacity was constrained. The greatest effects were seen when implementing multimodal strategies that simultaneously addressed buprenorphine initiation, duration, and capacity. These results demonstrate the importance of including treatment capacity and utilization in a care model to capture this dynamic.

The study results rely on the projections of SOURCE, which predicts changes in opioid use patterns and treatment demand.^18,19,57^ If the national demand for buprenorphine treatment decreases over time, as SOURCE predicts, interventions to expand capacity may only be needed in the next 4 to 6 years. If demand for buprenorphine treatment does not decrease, contrary to the SOURCE projections, or with the expansion of interventions associated with increased demand, hub-and-spoke interventions may be more effective and cost-effective than we have estimated, as it will increase capacity when it is constrained, especially when included in portfolios with interventions associated with increased treatment duration.

We demonstrated the effects of changes to buprenorphine treatment, only one of many potential options to treat OUD. In the main analysis, we considered a 10% coverage level of all interventions, demonstrating a potential to avert 8570 overdose deaths when implementing interventions. However, the study results suggest that even though the number of deaths averted was small, substantial QALYs are gained from strategies associated with increased buprenorphine treatment initiation, duration, and capacity. Furthermore, 10 strategies were cost-saving compared with the status quo in the societal perspective and 1 in the health care perspective, which shows that many combinations of interventions may improve health outcomes and avert more downstream costs than the initial investments required for the intervention.

### Limitations

This study was subject to limitations, including the limitations of the SOURCE model.^18,19^ We only included interventions associated with buprenorphine duration, capacity, and initiation based on quantitative evidence in the literature. Interventions may have multiple associations with buprenorphine use that were not captured in this analysis, making them potentially even more effective at increasing buprenorphine duration, capacity, and initiation than we have suggested and making the potential for health benefits even greater. Our analysis did not capture the mechanism by which each intervention affects buprenorphine use, and we did not highlight specific barriers that can be addressed by each intervention, such as financial, stigmatic, logistical, or regulatory barriers. This analysis was at the national level and did not consider regional, local, or demographic differences in OUD, buprenorphine capacity, or buprenorphine demand. Estimates of the effectiveness and costs of interventions were based on studies with a smaller scale than nationwide programs. There might be differences in the original study populations, efficiency gains, and scaling costs that we did not consider. Heterogeneity within each health state, including age and sex stratification, was not modeled. We did not compare the feasibility of implementing these strategies, although some may be more difficult to implement on a national scale than others. We lacked data on the interactions between interventions with which to compare model outcomes and how that might change costs and effects when interventions are implemented together. We did not include effects of interventions other than their association with buprenorphine treatment receipt (eg, psychotherapy’s association with mental health outcomes). An extension of this work would be to compare buprenorphine-specific interventions with additional OUD interventions, including methadone expansion, providing naloxone for harm reduction, and preventing addiction. Despite these limitations, this study offered potential insights to improve the models of care for OUD treatment.

## Conclusions

The study findings potentially add to the literature by considering how interventions affect the initiation, duration, and capacity of buprenorphine treatment using a system dynamics model, including capacity constraints. This is important, as capacity constraints can limit the effectiveness of interventions and even be associated with more deaths initially when increasing duration. The study results are consistent with previous studies that did not include capacity constraints, which found that increasing buprenorphine duration is cost-effective^11,13^ or cost-saving.^9^ However, this study suggested that the most benefit could be gained by implementing strategies that increase buprenorphine capacity and duration simultaneously. Additionally, we estimated that the benefits of increasing buprenorphine duration will be undermined unless capacity is expanded; otherwise, patients who want to initiate treatment may be crowded out by patients remaining in treatment longer.

## Notes

Supplement 1. **eMethods 1.** Overview of SOURCE model **eMethods 2.** Description of literature review process **eMethods 3.** Calculating final values for intervention effectiveness and costs **eMethods 4. **Methods for calculating expected end-of-life QALYs **eMethods 5. **Methods for calculating healthcare costs of opioid use disorder **eMethods 6. **Methods for calculating productivity and consumption costs **eMethods 7. **Sensitivity analysis process **eFigure 1. **SOURCE’s structure **eFigure 2.** Buprenorphine effective capacity utilization over time in the status quo **eFigure 3.** Cost-effectiveness acceptability curve **eTable 1.** Literature search systematic approach **eTable 2. **Effectiveness, retention, duration, and costs of contingency management (CM) in MOUD treatment studies **eTable 3. **Effectiveness, retention, duration, and costs of psychotherapy and counseling methods in MOUD treatment studies **eTable 4.** Provider training **eTable 5. **Emergency department initiation of buprenorphine **eTable 6. **Telemedicine for buprenorphine appointments **eTable 7. **Telemedicine costs for non-buprenorphine appointments **eTable 8. **Example model inputs and range for each intervention; 20% coverage level **eTable 9.** Sensitivity analysis - average incremental costs, QALYs gained, and net monetary benefit (NMB) by intervention compared to the status quo, 10% intervention coverage **eTable 10. **Expected intervention costs, QALYs, and cost per QALY gained for each strategy; healthcare perspective only, 10% coverage **eTable 11.** Expected overdose deaths for each strategy, with varying coverage interventions **eTable 12.** Expected intervention costs, QALYs, and cost per QALY gained for each strategy; 5% coverage of all interventions **eTable 13.** Expected intervention costs, QALYs, and cost per QALY gained for each strategy; 20% coverage of all interventions **eTable 14. **Quality-adjusted life years for each health state in SOURCE

Supplement 2. Data sharing statement
