# Supplementary material for: Cost-effectiveness of Increasing Buprenorphine Treatment Initiation, Duration, and Capacity Among Individuals Who Use Opioids
Source: JAMA Health Forum. 2023 May 19;4(5):e231080. doi: 10.1001/jamahealthforum.2023.1080 (PMC10199347; doi:10.1001/jamahealthforum.2023.1080)
Supplement: Supplement 1. — eMethods 1. Overview of SOURCE model eMethods 2. Description of literature review process eMethods 3. Calculating final values for intervention effectiveness and costs eMethods 4. Methods for calculating expected end-of-life QALYs eMethods 5. Methods for calculating healthcare costs of opioid use disorder eMethods 6. Methods for calculating productivity and consumption costs eMethods 7. Sensitivity analysis process eFigure 1. SOURCE’s structure eFigure 2. Buprenorphine effective capacity utilization over time in the status quo eFigure 3. Cost-effectiveness acceptability curve eTable 1. Literature search systematic approach eTable 2. Effectiveness, retention, duration, and costs of contingency management (CM) in MOUD treatment studies eTable 3. Effectiveness, retention, duration, and costs of psychotherapy and counseling methods in MOUD treatment studies eTable 4. Provider training eTable 5. Emergency department initiation of buprenorphine eTable 6. Telemedicine for buprenorphine appointments eTable 7. Telemedicine costs for non-buprenorphine appointments eTable 8. Example model inputs and range for each intervention; 20% coverage level eTable 9. Sensitivity analysis - average incremental costs, QALYs gained, and net monetary benefit (NMB) by intervention compared to the status quo, 10% intervention coverage eTable 10. Expected intervention costs, QALYs, and cost per QALY gained for each strategy; healthcare perspective only, 10% coverage eTable 11. Expected overdose deaths for each strategy, with varying coverage interventions eTable 12. Expected intervention costs, QALYs, and cost per QALY gained for each strategy; 5% coverage of all interventions eTable 13. Expected intervention costs, QALYs, and cost per QALY gained for each strategy; 20% coverage of all interventions eTable 14. Quality-adjusted life years for each health state in SOURCE [file jamahealthforum-e231080-s001.pdf]

Supplemental Online Content

Claypool AL, DiGennaro C, Russell WA, et al. Cost-effectiveness of increasing buprenorphine treatment initiation, duration, and capacity among individuals who use opioids. *JAMA Health Forum*. 2023;4(5):e231080. doi:10.1001/jamahealthforum.2023.1080

eMethods 1. Overview of SOURCE model

eMethods 2. Description of literature review process

eMethods 3. Calculating final values for intervention effectiveness and costs

eMethods 4. Methods for calculating expected end-of-life QALYs

eMethods 5. Methods for calculating healthcare costs of opioid use disorder

eMethods 6. Methods for calculating productivity and consumption costs

eMethods 7. Sensitivity analysis process

eFigure 1. SOURCE's structure

eFigure 2. Buprenorphine effective capacity utilization over time in the status quo

eFigure 3. Cost-effectiveness acceptability curve

eTable 1. Literature search systematic approach

eTable 2. Effectiveness, retention, duration, and costs of contingency management (CM) in MOUD treatment studies

eTable 3. Effectiveness, retention, duration, and costs of psychotherapy and counseling methods in MOUD treatment studies

eTable 4. Provider training

eTable 5. Emergency department initiation of buprenorphine

eTable 6. Telemedicine for buprenorphine appointments

eTable 7. Telemedicine costs for non-buprenorphine appointments

eTable 8. Example model inputs and range for each intervention; 20% coverage level

eTable 9. Sensitivity analysis - average incremental costs, QALYs gained, and net monetary benefit (NMB) by intervention compared to the status quo, 10% intervention coverage

eTable 10. Expected intervention costs, QALYs, and cost per QALY gained for each strategy; healthcare perspective only, 10% coverage

© 2023 Claypool AL et al. *JAMA Health Forum.*

eTable 11. Expected overdose deaths for each strategy, with varying coverage interventions

eTable 12. Expected intervention costs, QALYs, and cost per QALY gained for each strategy; 5% coverage of all interventions

eTable 13. Expected intervention costs, QALYs, and cost per QALY gained for each strategy; 20% coverage of all interventions

eTable 14. Quality-adjusted life years for each health state in SOURCE

This supplemental material has been provided by the authors to give readers additional information about their work.

© 2023 Claypool AL et al. *JAMA Health Forum.*

eMethods 1: Overview of SOURCE model

SOURCE (1,2) tracks opioid misuse, including OUD involving prescription opioids and heroin. SOURCE models the three FDA-approved MOUDs: methadone, buprenorphine, and extended-release injectable naltrexone. Each medication varies in the average duration of treatment, treatment capacity, and treatment-seeking rate. In SOURCE, mortality is based on the type of opioid use, likelihood of fentanyl exposure associated with heroin use, probability of naloxone administration, treatment status, and remission status.

Dynamic feedback loops are a key feature of SOURCE and of the cost-effectiveness analysis as they capture the interactions between the supply of and demand for MOUD. These feedback loops affect transition rates in the model and include social influence factors, risk perception, capacity limits on treatment, and availability of prescription and illicit opioids. For instance, capacity limits on buprenorphine prescribers and other systemic barriers to prescribing reflect real-life limits of buprenorphine provision (3).

SOURCE includes additional time-dependent trends as inputs. For example, SOURCE reflects that fentanyl contamination in the heroin supply as well as the availability of naloxone, an opioid overdose reversal medication, have increased over time. These trends affect the risk of mortality in the event of an opioid-related overdose. SOURCE does not consider overdose deaths that are unrelated to intentional opioid use and does not specify the number of overdoses resulting from fentanyl contamination of non-opioid substances. SOURCE was calibrated to data (1999-2020), including target data from the National Survey on Drug Use and Health, the National Vital Statistics System, and IQVIA prescription data—see (1,2).

In SOURCE, buprenorphine reduced the hazard of overdose death while in treatment (2). Also, the average duration is linked to treatment success based on the assumption that the longer the treatment duration, the greater the probability of exiting treatment into remission rather than returning to OUD.

eFigure 1 presents the overview of SOURCE. Details about data inputs, assumptions, and validation are reported elsewhere (1,2).

eMethods 2: Description of literature review process

For each intervention, the review included specific search terms and processes (eTable 1). Two researchers (AFZ, ZR) conducted a review of the titles and abstracts, completed a full-text review of the papers that met the search criteria, extracted results on effectiveness and costs for each intervention, and discussed their results with a third researcher (ALC).

After reviewing the titles of all initial search results, we reviewed 180 titles and abstracts, and 45 complete manuscripts (eTable 1). We aggregated the quantitative results for the effectiveness of interventions based on trials, retrospective data review, and modeling studies and presented them in tables to compare similar values (eTables 2-6). Title and abstract reviews were conducted in Rayyan systematic review software (4).

The values selected for the base-case effectiveness for each intervention are highlighted below.

© 2023 Claypool AL et al. *JAMA Health Forum.*

Inclusion Criteria for the literature search included:

- Quantitative measures of policy- effectiveness or costs
- Treatment for opioid use disorder included in the study
- Clearly about opioid use disorder
- Contains buprenorphine prescribed for opioid use disorder (OUD)
- Intervention was the primary part of the intervention
- Includes ages ≥ 18
- Peer-reviewed journal article
- In English or translated to English

eMethods 3: Calculating final values for intervention effectiveness and costs

Intervention effectiveness
We selected the final values for the effectiveness of interventions based on whether we could implement these findings into the variables of SOURCE and the robustness of methods for estimating the effectiveness of interventions before and after implementation. For example, Fairley et al. (5) conducted a meta-analysis to calculate the hazard rate ratios of contingency management (HR=0.594) and psychotherapy (HR=0.986) and included annual costs (5). For the hub and spoke model, we used the Vermont hub and spoke results for our effectiveness measures because they studied the change in the number of prescribers and the number of patients per prescriber before and after program implementation (6). To model the effectiveness of ED initiated buprenorphine, we considered the percent of people who were still enrolled in buprenorphine after 30 days in four studies (7–10) and compared those to the control 30-day treatment retention in D'Onofrio to calculate a mean, minimum, and maximum treatment-seeking ratio of ED initiation (1.556 [1.164, 2.108]). ED initiation effectiveness was applied to the number of people in the ED, which we estimated as the percent of people per year with OUD involving prescription opioids and/or heroin (with a model-estimated probability of exposure to fentanyl) who would experience a non-fatal opioid overdose. This was estimated to be 26% in the SOURCE model run of the status quo.

The total number of people to receive the ED initiation intervention at time *t* is modeled as:

$$N_{ED}^{t} = c(P(nfod|OUD)N_{OUD} + P(nfod|HUD)N_{HUD})$$

N_{ED}^{t} = *number of people who receive ED-initiated buprenorphine at time t*

$$c = coverage\ rate\ of\ intervention\ in\ ED$$

$$P(nfod|OUD) = \ yearly\ probability\ of\ nonfatal\ overdose\ given\ OUD$$

$$P(nfod|HUD) = \ yearly\ probability\ of\ nonfatal\ overdose\ given\ HUD$$

$$N_{OUD} = Population\ of\ people\ with\ OUD\ in\ SOURCE$$

$$N_{HUD} = Population\ of\ people\ with\ HUD\ in\ SOURCE$$

Increase in average duration of buprenorphine prescribing while in telehealth was calculated using the average duration with and without telehealth in the Vakkalanka study.

Intervention costs

Annual costs for contingency management services and psychotherapy were derived in Fairley, et al. (5). We calculated the cost of buprenorphine per provider ($31,593, 2017 USD), by dividing the Washington-state hub and spoke program maximum annual contract per network ($789,825, 2017 USD) by the average number of providers per network (n=25) and adjusting for inflation (12). For ED initiation, we summed the screening cost ($214, 2017 USD) and intervention costs ($83, 2017 USD) from Busch et al. 2017 (13) plus a cost of additional counseling services (14) ($225, 2020 USD), meant to represent a bridge program from buprenorphine in the ED to linkage with a longer-term provider. We did not find the cost of telehealth for buprenorphine prescribing. However, for all results of cost of telehealth for other types of appointments, the cost of a telehealth appointment was less than for in-person appointment (eTable 7). Therefore, we assume the cost of telehealth is $0 compared to in-person appointments. We did not consider fixed technology installment costs, which is consistent with other cost-effectiveness analyses (15). Additional, initial costs for scale-up of interventions were not included. All costs were adjusted to 2021 U.S. Dollars using the Consumer Price Index.

eMethods 4: Methods for calculating expected end-of-life QALYs

For opioid overdose deaths, we calculated the estimated lifetime QALY lost by applying age-specific mean quality of life values estimated in Sullivan and Ghushchyan 2006 (16) to the expected remaining life years based on the age-specific annual probability of death according to CDC life tables. We discounted future QALYs at 3% annually. The age-weighted average of end-of-life QALYs was calculated using the estimated age-stratified population of people with OUD in the United States (17).

eMethods 5: Methods for calculating healthcare costs of opioid use disorder

Background healthcare costs

We used average baseline healthcare cost estimates from Medical Expenditure Panel Survey (MEPS) (18) The excess healthcare cost of the population with OUD is obtained from (19) separately for the treated and untreated subpopulations and without including the treatment cost of OUD. We conservatively assumed that patients with OUD who are receiving treatment have 20% higher costs on average than those not receiving treatment, which also aligns with other studies in the literature (5,19,20).

Cost of non-fatal and fatal opioid overdose

Opioid overdose cost consists of the expected costs of calling Emergency Medical Services (EMS) to transport to a hospital, ED visit, and hospital stays. We assumed that 90% of the opioid overdose cases are transported to the hospital by EMS (21), and the costs of calling EMS and transport to the hospital were obtained from (21–23). We estimate that 73% of non-fatal opioid overdoses are discharged from ED versus the remaining are discharged after inpatient stays (21). Whereas 1.4% of fatal opioid overdoses are observed in ED and 6.2% during an inpatient stay

(24). Varying levels (low, medium, high) of cost estimates for ED (21,22,25) inpatient stay (21,22,24) and EMS (21–23) are obtained from the literature.

MOUD costs
Methadone treatment: Treatment cost includes the cost of methadone and associated drug administration cost. We assumed that methadone is administered daily for patients' treatment programs.

Buprenorphine treatment: Treatment cost includes the cost of buprenorphine, drug monitoring, and counseling services. We assumed that the patient would receive medication counseling twice a week.

Naltrexone treatment: Treatment cost includes the cost of injectable naltrexone, administration, and related services (e.g., drug monitoring, counseling services, etc.). We assumed that the patient would administer naltrexone and receive medication counseling services once per month.

eMethods 6: Methods for calculating productivity and consumption costs

Expected remaining life years lost due to overdose
Following the recommendations of the Second Panel on Cost-Effectiveness in Health and Medicine (26) we included the loss of future productivity minus their future consumption as their net productivity loss for each overdose death. We first calculated the age-specific expected remaining life years lost using CDC life tables (27)

Productivity
We used the mean income by age group as estimated in the U.S. Census Bureau, Current Population Survey, 2020 Annual Social and Economic Supplement (CPS ASEC) (28).

Consumption
We used the age group-specific mean expenditure per person as estimated in the Consumer Expenditure Survey, U.S. Bureau of Labor Statistics from September 2020 (29) To calculate the net present value, we calculated net productivity (productivity minus consumption) for each year, multiplied by the probability an individual would have lived to that year from the life tables and discounted all future values by 3%.

Age-weighted average
Age-weighted averages for consumption and productivity were calculated using the estimated age-stratified population of people with OUD in the United States (17) and adjusted for inflation to reflect 2021 USD using the Consumer Price Index. We assumed productivity and consumption costs were equal for individuals experiencing OUD and those not experiencing OUD, similar to Appendix A in the Second Panel on Cost-Effectiveness in Health and Medicine (30).

eMethods 7: Sensitivity analysis process

We conducted a probabilistic sensitivity analysis of intervention effectiveness by running 1,000 simulations for each intervention or combination of interventions using effectiveness

© 2023 Claypool AL et al. *JAMA Health Forum.*

parameters and intervention and treatment costs, sampled from uniform distributions over the uncertainty ranges, when available (Table 1, eTable 8). For input and treatment costs, we assumed a range that was 25% above and below each basecase value.

We calculated the net monetary benefit (NMB) for each run for intervention to consider if they were considered cost-effective compared to the status quo (NMB > 0).

$$NMB = Incremental\ QALYs\ \times WTP - Incremental\ Costs$$

We then reviewed the percent of runs in which each intervention or combination of interventions was considered cost-effective compared with the status quo of no additional interventions as well as the percent of runs in which each intervention was the preferred intervention (NMB the highest for the intervention compared all other interventions for that simulation run).

© 2023 Claypool AL et al. *JAMA Health Forum.*

Supplemental figures

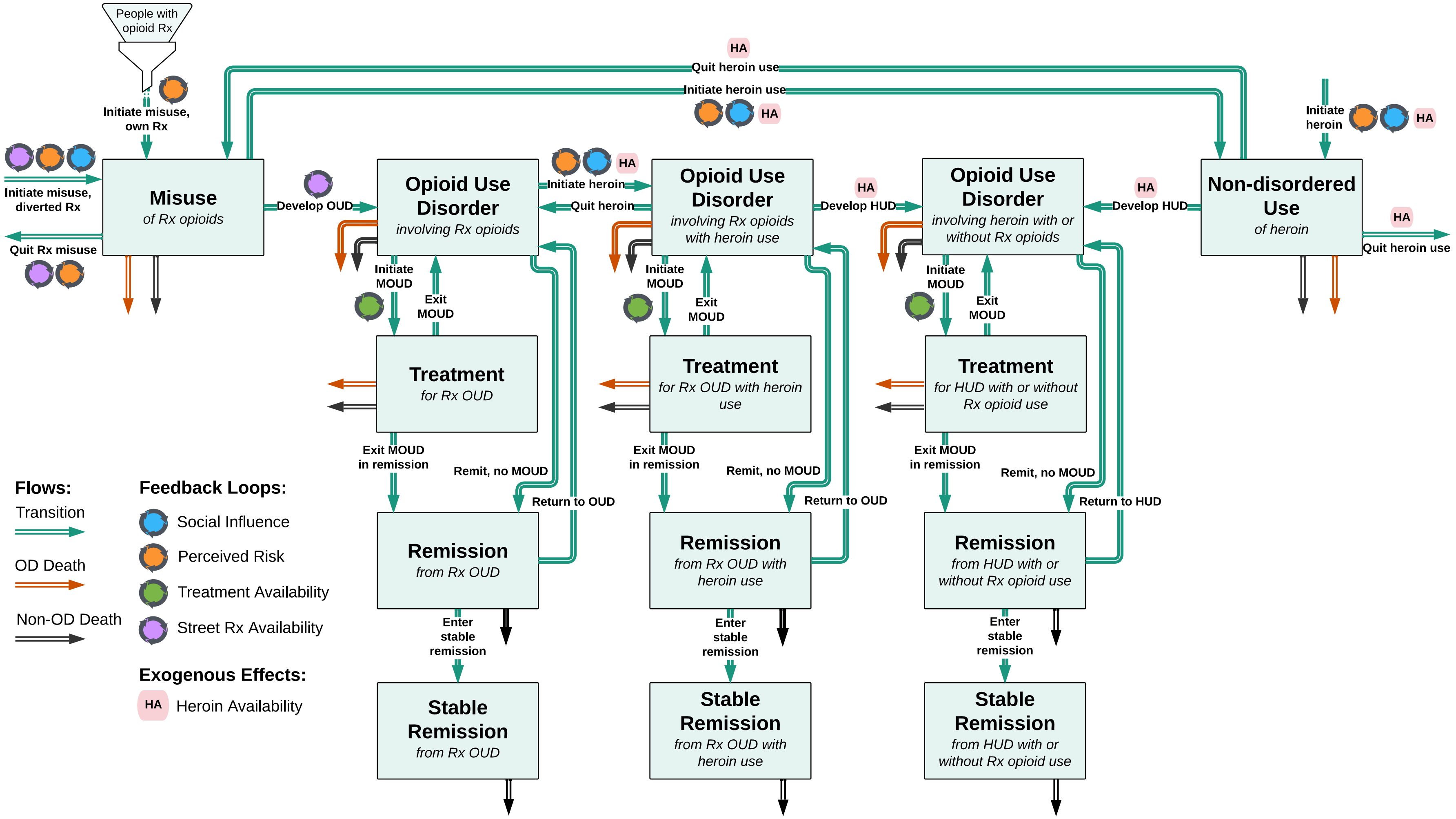

eFigure 1: SOURCE’s structure (2)

© 2023 Claypool AL et al. *JAMA Health Forum.*

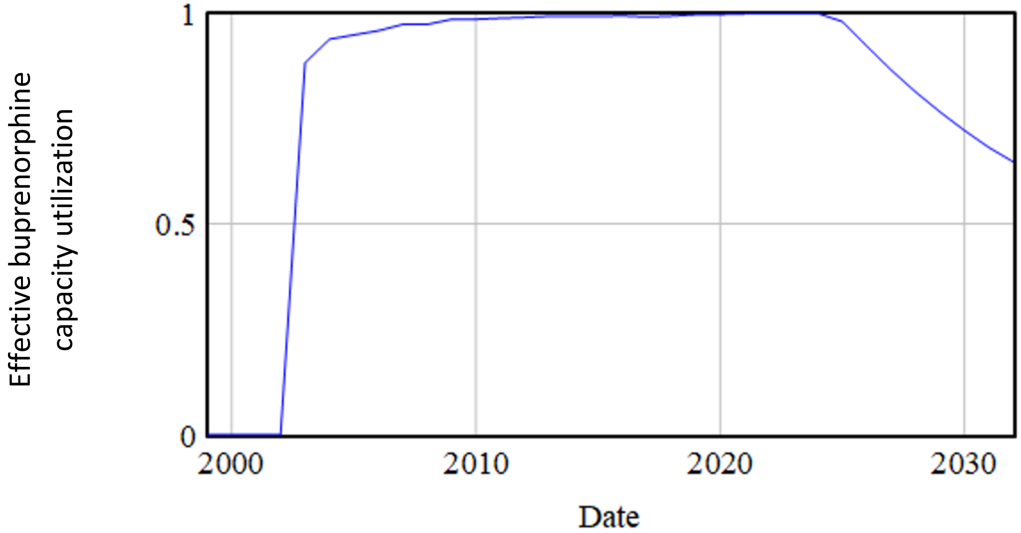

eFigure 2: Buprenorphine effective capacity utilization over time in the status quo
Effective capacity reflects the number of buprenorphine providers and the average number of patients that each provider sees. Effective capacity reflects the impacts of provider-side barriers on their willingness and ability to prescribe buprenorphine. This may include regulatory, logistical, and stigmatic barriers.

© 2023 Claypool AL et al. *JAMA Health Forum.*

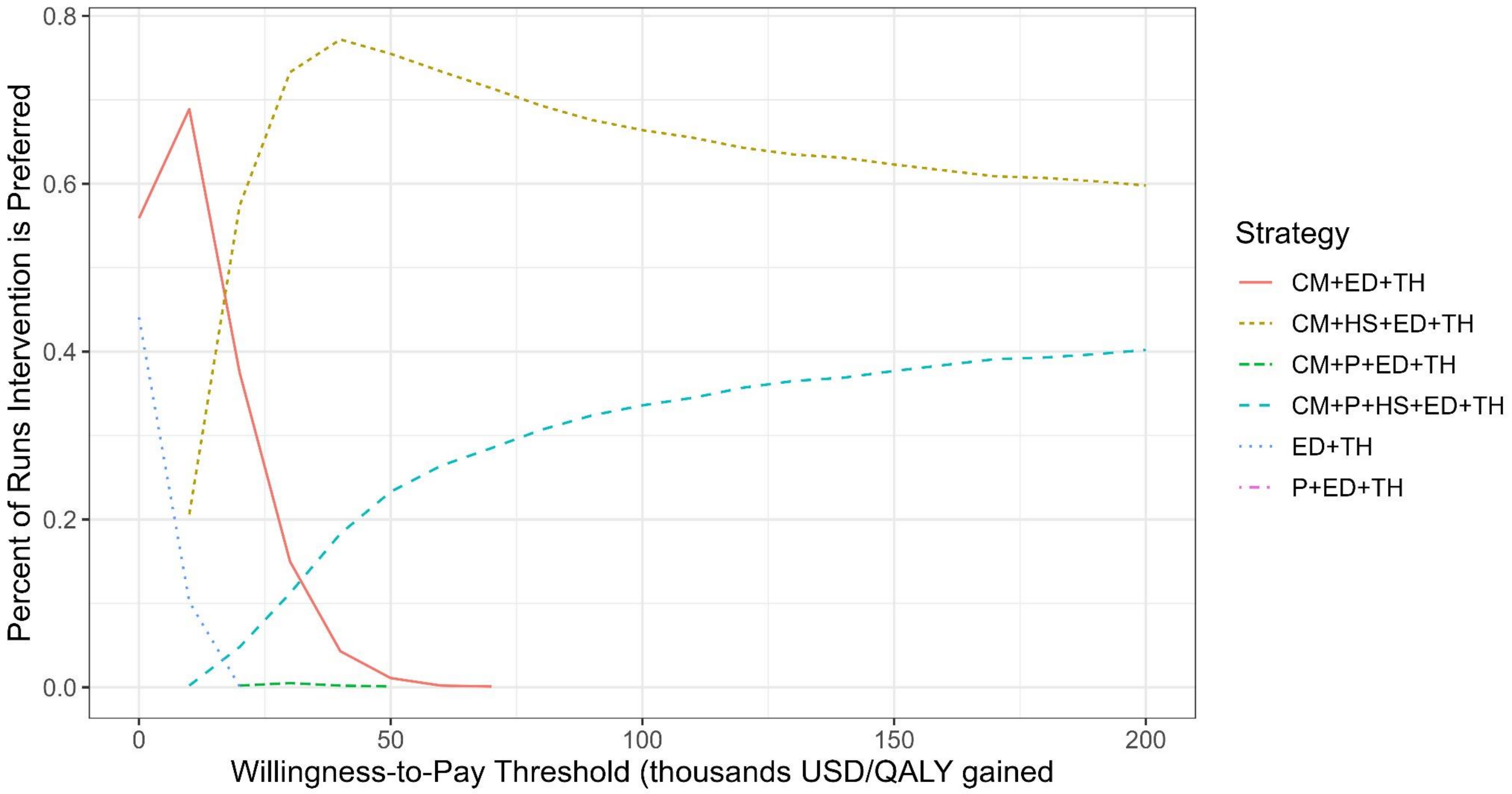

eFigure 3: Cost-Effectiveness Acceptability Curve

Each line represents a single strategy of the sensitivity analysis in which intervention effectiveness parameter(s). Out of the 1000 Probabilistic Sensitivity Analysis (PSA) runs, it shows the percent of runs where each strategy was the preferred strategy, for willingness to pay thresholds from $0-$200,000/ QALY gained. Strategies that were never preferred are excluded.

© 2023 Claypool AL et al. *JAMA Health Forum.*

Supplemental tables

eTable 1: Literature search systematic approach

Topic	Dates of Search*	Search Terms Used (All)	Number of Search Results	Number of Titles and Abstracts Revised	Number of Full Papers Read	Final Number of Papers Included [o]
Contingency Management	5/1/21 – 7/1/21	contingency management in methadone clinics; opioid use disorder contingency management; prize reinforcement opioid use; contingency management in opioid clinics; contingency management for opioids	749	38	11	2
Psychotherapy and Counseling	7/6/21 – 12/1/21	psychotherapy medications for opioid use disorder, psychotherapy opioid use, counseling opioid use disorder, counseling buprenorphine/methadone/naltrexone, therapy and opioid use disorder, maintenance therapy, - opioid maintenance+ group/clinic therapy	63	42	9	7
Hub and Spoke, Provider Training	5/19/21 – 7/1/21	opioid use disorder, training, capacity, treatment, buprenorphine, MOUD, OUD, medications, provider, physician, waiver, education, cost, cost-effectiveness, duration	900	100	25	15
ED initiated Buprenorphine	11/10/21 – 4/1/22	emergency department, ED, initiation, buprenorphine, opioid use, cost	131	131	28	14
Telehealth	1/18/22 – 2/10/23	telemedicine, telehealth, buprenorphine, opioid use disorder, cost	185	125	41	15

*A combination of PubMed and Google Scholar was used. [o] Final papers limited to buprenorphine studies only.
MOUD=Medication for opioid use disorder; OUD=Opioid use disorder; ED=Emergency Department

© 2023 Claypool AL et al. *JAMA Health Forum.*

eTable 2: Effectiveness, retention, duration, and costs of contingency management (CM) in MOUD treatment studies

Source	Type of MOUD	Type of CM Studied	Number of Patients		Duration of Study (Months)	Duration of CM (Months)	Average treatment retention (weeks)		Percent of Negative Urine Samples		Hazard Ratio of discontinuation	Average Cost of CM per treatment	Time Frame of Costs (Months)	Costs Included
			ST	CM			ST (sd)	CM (sd)	ST (sd)	CM (sd)				
Fairley et al. (5)	Methadone, buprenorphine, or XR-naltrexone	Prize based & Voucher Based	-	-	-	12	-	-	-	-	0.594 (0.437-0.787)	$3385 (3055-3732)	12	VA data of cost of CM per patient in treatment
Ling et al. (31)	Buprenorphine	Prize based	51	49	4.5	4	14.6 (5.1)	14.6 (5.3)	10.86 (10.7)	14.04 (12.3)	-	$230-$1460	4	Costs of prizes

ST=Standard Treatment, CM=Contingency Management, VA=U.S. Department of Veterans Affairs

© 2023 Claypool AL et al. *JAMA Health Forum.*

eTable 3: Effectiveness, retention, duration, and costs of psychotherapy and counseling methods in MOUD treatment studies

Source	Type of MOUD	Duration of Study (Months)	Duration of Therapy (Months)	Type of Therapy Studied	Longest Duration of Abstinence (LDA) in weeks		Average Duration (months)		Mean Rate Negative Urine Samples		Hazard Ratio of Discontinuation	Cost of Therapy per treatment (USD)	Time Frame of Costs (Months)	Costs Included
					Standard Medical Mngt	Enhanced Medical Mngt	Standard Medical Mngt	Enhanced Medical Mngt	Standard Medical Mngt	Enhanced Medical Mngt				
Fiellin et al. 2006 (32)	Buprenorphine-naloxone	6	6	Standard medical management: (manual guided, medically focused counseling) Enhanced medical management: (manual guided, medically focused counseling extended)	1.675	1.375	4.3	4.5	0.4	0.40	-	-	-	-
Fairley et al. 2021 (5)	Methadone, buprenorphine, or XR-naltrexone	-	12	-	-	-	-	-	-	-	0.986 (0.772-1.240)	4295 (range 3877-4736)	12	Psychotherapy, 2019 USD, annual treatment cost
Polsky et al. 2010 (33)	Buprenorphine	15	3	Twice weekly drug counseling	-	-	-	-	-	-	-	Individual counseling: 27<br>Group: 9<br>Family: 30	12	Cost of counseling
Tacke et 2009 al. (34)	Buprenorphine-naloxone	1	1	Weekly visits for supervised drug administration and counseling.	-	-	-	-	-	-		4502	1	Cost of therapy + buprenorphine-naloxone, travel costs to clinic, price of Pharma DDS-I package, packaging costs

© 2023 Claypool AL et al. *JAMA Health Forum.*

eTable 4: Provider training

Source	Population	Type of Intervention	# of additional waivered physicians	# of additional waivered NPs/PAs	# of additional patients treated	# of patients per physician	% providing/prepared to provide buprenorphine treatment
Brooklyn et al., 2017 (6)	Vermont OUD providers/patients	In-person: Hub and Spoke	110 (64% increase)		1900 (211% increase)	50% increase	
Winstanley et al., 2020 (35)	West Virginia OUD providers/patients	In-person: Hub and Spoke	56		196		
Levin et al., 2016 (36)	U.S. physicians participating in the program	Online: Providers' Clinical Support System for Medication Assisted Treatment (PCSS-MAT)	4130				
Komaromy et al., 2016 (37)	New Mexico primary care providers	Online: Project ECHO	275				
Auty et al., 2020 (38)	U.S. physicians, Nurse Practitioners, and Physician Assistants	Waiver training		28,010			
Tong et al., 2018 (39)	Certified family doctors who graduated residency in 2013	Training during residency					10% prepared to provide buprenorphine treatment, 7% current providing buprenorphine treatment
Wen et al., 2018 (40)	Medicaid prescriptions	Waiver training					10% increase in buprenorphine prescribing rate associated with 10% increase in waivered physicians
Foster et al., 2020 (41)	3 EDs in Philadelphia	In-person	Increased from 6% to 90%				Prescribing rate of <1% increased to 15%
Stokes et al., 2021 (42)	National Modeling study to find the theoretical maximum prescribers that could be added	In-person, medical school waiver training			532,890 per year		

© 2023 Claypool AL et al. *JAMA Health Forum.*

eTable 5: Emergency department initiation of buprenorphine

**Source**	**Intervention**	**Study Type**	**Treatment after 30 days (intervention)**	**Treatment after 30 days (control)**
D'Onofrio et al. (2015) (7)	ED initiation	RCT	78% [70%-85%]	37% [28%-47%]
Jennings et al. (2021) (8)	ED initiation	Retrospective review	43.1%	
Kaucher et al. (2020) (9)	ED initiation	Retrospective review	49.3%	
Bogan et al. (2020) (10)	ED initiation	Prospective study	59.9%	
		**Average:**	**57.6%**	

Average relative rate of treatment seeking = 57.56/37.0

Minimum relative rate of treatment seeking = 43.1/37.0

Maximum relative rate of treatment seeking = 78.0/37.0

© 2023 Claypool AL et al. *JAMA Health Forum.*

eTable 6: Telemedicine for buprenorphine appointments

Source	Number of Patients		Duration of Study (months)	Average retention in treatment (days)		Percent Retained in Treatment (%)	
	ST	TM		ST	TM	ST	TM
Lin, Fortney, et al. 2022 (43)	30,898	2,718	84	295	722 (322-1459)	-	-
Weintraub et al. 2021 (44)	24	94	3	-	-	58.47	58.51
Belcher et al. 2021 (45)	-	7	3	-	21	-	-
Weintraub et al. 2018 (46)	-	177	3	-	-	-	57.4
Harris et al. 2022 (47)	131	19	12			82.4	68.4
Tofighi et al. 2022 (48)	-	78	3	-	-	-	53.8
Rahman et al. 2021 (49)	-	85	3	-	-	-	98.7
Yeo et al. 2021 (50)	166	111	6	-	-	91.5	51.9
Ruetsch et al. 2012 (51)	439	987	12	-	-	56.1	67.7
Sahu et al. 2022 (52)	487	811	24			61	49.3
Vakkalanka et al. 2022 (53)*	30270	593	120	Discontinuation hazard rate ratio of telehealth= 0.66 (0.60, 0.78)			
Samuels et al. 2022 (54)	0	159	4				83.05%
Lin, Zhang, et al. 2022 (55)	0	15,339	12	203.8 (pre-pandemic)	208 days (pandemic)		
Kaur et al. 2022 (56)	0	56,000	12				Group 1: 92.55%, Group 2: 94.18%, Group 3: 90.83%, Group 4: 95%
Frost et al. 2022 (57)	2,111	15,835	12			32.2% (post-COVID-19)<br><br>51.1% (pre-COVID-19)	25.4% (post-COVID-19)<br><br>43.0% (pre-COVID-19)

*Vakkalanka et al. was chosen as the source for telehealth effectiveness because it was a longitudinal study that considered the full duration of buprenorphine treatment when exposed to telehealth. Lin, Fortney, et al. 2022 was not used in the estimate because it was a descriptive results, and Lin, Zhang, et al. 2022 was not used because it evaluated the differences before and after March 2020 to study the differences in treatment during the pandemic. All other studies were not included because they only reported on percent retained in treatment over a certain time period, which could not produce an input for SOURCE.

© 2023 Claypool AL et al. *JAMA Health Forum.*

eTable 7: Telemedicine costs for non-buprenorphine appointments

Study	Type/field of study	Type of Costs	Cost of telemedicine appointment	Cost of in-person appointment	Cost of telehealth compared to in-person	Type and year of currency
Xu et al. 2008 (58)	ear, nose, and throat consultations	per consultation for telehealth	108	155	-47	US (2019)
Armstrong et al. 2007 (59)	store-and-forward system for dermatology screening	hourly operating costs of care	361	456	-95	US (2019)
Pare et al. 2006 (60)	monitoring for patients with chronic obstructive pulmonary disorder	total service cost per treatment	8566	13713	-5147	US (2019)
Labiris et al. 2005 (61)	videoconference consultations mainly for orthopedics and dermatology	cost per consultation	327	333	-6	US (2019)
Scuffham et al. 2002 (62)	dentist videoconference	per treatment	233	662	-429	US (2019)
Bergmo et al. 2000 (63)	store-and-forward system for dermatology screening	per patient per year	96042.79	179634.98	-83592.19	US (2019)
McCue et al. 1998 (64)	review of triage of specialists cases (HIV, cardiology, and oral surgery)	per visit	430	835	-405	US (2019)
Frederix et al. 2016 (65)	monitoring for cardiovascular disease	per treatment	2156	2720	-564	Euro (2015)
Greving et al. 2015 (66)	monitoring for vascular disease using patient-collected biometric information	per treatment	4859	5078	-219	Euro (2009)
Nguyen et al. 2016 (67)	store-and-forward system for diabetic retinopathy screening	per treatment	1914	2059	-145	Singapore $ (2015)
Thomas et al. 2015 (68)	store-and-forward ophthalmic images for glaucoma screening	per treatment	872	4441	-3569	Canada $ (2014)
Zanaboni et al. 2013 (69)	monitoring of biometric data from an implanted device to identify heart failure exacerbations	per treatment	291.36	381.34	-89.98	Euro (2010)

© 2023 Claypool AL et al. *JAMA Health Forum.*

eTable 8: Example model inputs and range for each intervention; 20% coverage level

Intervention	Transition in Model (as seen in eFigure 1)	Parameter in model	Coverage level	Base case	Lower bound	Upper bound	Calculation of base case*	Source
ED Initiated Buprenorphine	Initiate MOUD (buprenorphine) for HUD	Buprenorphine treatment seeking rate - HUD	20% of people with HUD that experience a non-fatal overdose°	1.35	1.32	1.39	0.2*0.26*1.31*1.556+ (1-(0.2*0.26))*1.31	(7–10)
	Initiate MOUD (buprenorphine) for OUD	Buprenorphine treatment seeking rate - OUD	20% of people with OUD that experience a non-fatal overdose°	0.317	0.311	0.326	0.2*0.26*0.308*1.556+ (1-(0.2*0.26))*0.308	(7–10)
Contingency Management	Exit MOUD (buprenorphine) in remission or not in remission	Average duration of buprenorphine treatment (years)	20% of buprenorphine patients	0.693	0.64	0.77	0.8*0.61+0.2*(0.61/0.594)	(5)
Psychotherapy	Exit MOUD (buprenorphine) in remission or not in remission	Average duration of buprenorphine treatment (years)	20% of buprenorphine patients	0.612	0.59	0.65	0.8*0.61+0.2*(0.61/0.986)	(5)
Telehealth	Exit MOUD (buprenorphine) in remission or not in remission	Average duration of buprenorphine treatment (years)	20% of buprenorphine patients	0.66	0.60	0.78	0.8*0.61+0.2*(0.61/0.69)	(53)
Hub and Spoke	Initiate MOUD (based on treatment availability)	Total number of buprenorphine providers	20% of buprenorphine prescribers	12.8% increase	5% increase	20% increase	0.64*0.2	(6)
	Initiate MOUD (based on treatment availability)	Decay constant of buprenorphine provider capacity	20% of buprenorphine prescribers	4.005E-5	3.56E-5	4.2275E-5	(1-(0.5*0.2))*4.45E-5	(6)
Contingency Management + Psychotherapy	Exit MOUD (buprenorphine) in remission or not in remission	Average duration of buprenorphine treatment (years)	20% of buprenorphine patients	0.696	0.61	0.85	0.8*0.61+0.2*(0.61/(0.594*0.986))	(5)

*Average duration of buprenorphine for no intervention is 0.61 years. ° Non-fatal overdose rate assumed to be 26% per year, according to SOURCE runs.

© 2023 Claypool AL et al. *JAMA Health Forum.*

eTable 9: Sensitivity analysis - average incremental costs, QALYs gained, and net monetary benefit (NMB) by intervention compared to the status quo, 10% intervention coverage

Strategy	Average Incremental QALYs compared to the Status Quo	Lower bound-Incremental QALYs	Upper bound-Incremental QALYs	Average Incremental Costs Compared to the Status Quo (USD 2021)	Lower bound-Incremental Costs (USD 2021)	Upper bound-Incremental Costs (USD 2021)	Average NMB Compared to the Status Quo* (USD 2021)	Lower bound-NMB* (USD 2021)	Upper bound-NMB* (USD 2021)	Percent of Runs that are Cost-Effective Compared to Status Quo* (%)	Percent of Runs where intervention is preferred*
CM	171,567	79,652	294,566	-403,839,522	-17,142,295,270	16,317,013,785	17,560,570,124	-7,501,984,726	43,842,875,707	96	0
P	3,062	-61,230	85,772	2,308,023,380	-14,029,419,429	18,949,943,082	-2,001,853,309	-23,306,433,326	21,808,958,566	41.7	0
HS	87,286	42,555	127,545	4,508,765,209	-12,840,907,978	21,231,684,850	4,219,789,836	-14,474,557,210	24,503,021,380	70.6	0
ED	101,301	26,831	176,253	-618,242,668	-17,975,608,225	16,027,360,840	10,748,345,316	-11,049,763,848	34,955,040,418	88.8	0
TH	131,004	82,697	178,384	-1,461,708,769	-18,346,763,627	15,185,704,397	14,562,130,355	-6,477,933,976	34,785,476,626	97.3	0
CM+P	173,276	29,282	325,535	1,641,046,919	-15,440,818,417	18,555,811,817	15,686,552,202	-10,185,176,455	44,802,953,673	92.5	0
CM+ED	261,114	109,583	424,731	-1,204,484,013	-17,597,102,011	15,594,614,234	27,315,879,535	-2,298,064,327	58,511,027,091	99.7	0
CM+ED+TH	344,574	191,099	478,294	-2,508,757,359	-19,044,445,479	14,311,226,848	36,966,195,380	7,614,863,883	64,343,861,971	100	0
CM+HS	307,019	133,290	564,704	3,358,639,976	-14,860,049,060	21,188,752,678	27,343,265,691	-5,399,750,443	63,607,640,488	99.7	0
CM+HS+ED	410,714	181,451	733,779	2,426,340,849	-15,586,152,760	20,394,129,850	38,645,092,314	155,639,251	78,998,936,044	100	0
CM+HS+ED+TH	543,127	265,792	873,427	658,732,298	-17,392,843,570	18,206,199,693	53,653,933,768	12,422,202,057	91,041,740,492	100	66.4
CM+P+HS	310,443	85,093	645,638	5,393,724,194	-13,156,790,371	23,443,221,708	25,650,574,275	-6,034,476,753	61,155,840,816	97.9	0
CM+P+ED	262,675	69,032	436,089	844,972,467	-16,066,493,268	17,711,945,899	25,422,562,179	-5,275,003,610	57,612,555,632	99	0
CM+P+HS+ED	413,939	134,340	761,359	4,467,333,347	-13,897,735,697	22,650,483,583	36,926,611,415	-2,862,150,142	77,484,158,308	99.9	0
CM+P+HS+TH	448,086	186,862	776,353	3,591,367,309	-14,518,104,455	21,221,096,935	41,217,259,647	8,435,370,789	81,006,296,690	100	0
CM+P+TH	264,722	112,203	382,875	274,978,884	-16,355,680,781	17,091,219,042	26,197,200,940	873,368,500	50,454,321,018	100	0
CM+P+ED+TH	345,472	163,141	481,868	-444,531,531	-16,874,839,113	16,428,763,995	34,991,711,609	6,029,258,043	63,199,943,593	100	0
CM+TH	263,659	157,775	367,460	-1,784,964,514	-18,284,554,153	14,973,564,519	28,150,817,299	2,800,335,538	51,596,173,743	100	0
CM+HS+TH	445,498	234,614	712,939	1,536,819,184	-16,544,745,728	18,963,740,808	43,012,997,018	7,084,388,044	84,989,288,430	100	0
ED+TH	223,868	113,811	330,815	-2,294,749,730	-19,739,954,330	14,443,228,144	24,681,577,973	-1,248,809,372	51,422,143,805	99.9	0
HS+ED	196,491	76,893	317,371	3,524,644,759	-14,137,903,456	20,173,433,050	16,124,437,516	-9,706,729,732	44,384,502,670	95.7	0

© 2023 Claypool AL et al. *JAMA Health Forum.*

HS+ED+TH	354,448	168,276	537,676	1,511,296,815	-16,417,079,089	18,246,190,276	33,933,501,702	1,605,477,058	64,402,783,604	100	0
P+HS	91,547	-20,703	232,696	6,517,331,770	-10,804,768,626	23,782,923,830	2,637,363,356	-22,055,968,966	31,140,464,545	60.8	0
P+HS+ED	200,657	29,653	402,635	5,538,460,605	-11,677,717,081	22,614,362,366	14,527,256,127	-14,842,172,149	48,824,311,435	91.5	0
P+HS+TH	252,316	87,288	435,798	4,493,569,308	-12,671,756,530	21,330,180,898	20,738,031,395	-8,237,036,985	52,044,210,008	98.5	0
HS+TH	248,605	135,749	359,812	2,466,323,044	-14,743,566,046	19,398,974,967	22,394,204,841	-3,796,740,218	48,442,540,471	99.5	0
P+ED	104,165	(28,784)	243,114	1,403,510,533	-15,585,577,575	18,145,482,480	9,013,002,384	-16,030,593,426	36,060,900,489	83.2	0
P+ED+TH	225,677	65,081	365,825	-251,571,627	-17,398,157,401	16,560,784,527	22,819,230,780	-3,753,327,563	48,449,766,233	99.6	0
P+HS+ED+TH	357,994	123,872	590,498	3,544,284,926	-13,964,288,102	20,389,190,002	32,255,120,508	-1,042,878,605	68,340,714,363	99.9	0
P+TH	132,903	31,289	237,080	577,384,580	-15,983,542,354	17,304,117,326	12,712,934,366	-9,291,865,263	37,746,758,971	92.6	0
All interventions	545,504	225,776	889,505	2,718,849,098	-15,068,315,618	20,464,128,481	51,831,538,684	12,703,867,597	96,095,729,366	100	33.6

*Net monetary benefit (NMB) calculated assuming a $100,000 per QALY willingness-to-pay threshold. SQ=Status Quo; CM=Contingency Management; P=Psychotherapy; HS=Hub and Spoke; ED=Emergency Department Initiation; TH=Telehealth

© 2023 Claypool AL et al. *JAMA Health Forum.*

eTable 10: Expected intervention costs, QALYs, and cost per QALY gained for each strategy; healthcare perspective only, 10% coverage

Strategy*	Total QALYs	QALYs Gained Compared to SQ	Total Cost (2021 USD)	Incremental Cost Compared to SQ (2021 USD)	ICER** (2021 USD per QALY gained)
ED	236,022,155	89,696	1,022,377,232,892	-5,136,093	ND
SQ	235,932,459	0	1,022,382,368,985	0	D
ED+TH	236,142,017	209,558	1,022,404,071,916	21,702,931	224
TH	236,059,326	126,866	1,022,415,609,629	33,240,644	D
CM+ED+TH	236,273,859	341,400	1,024,026,594,681	1,644,225,696	12,307
CM+TH	236,202,407	269,948	1,024,044,899,592	1,662,530,607	D
CM+ED	236,193,233	260,774	1,024,051,305,445	1,668,936,460	D
CM	236,114,587	182,127	1,024,064,101,085	1,681,732,100	D
P+ED	236,026,188	93,729	1,024,455,911,070	2,073,542,085	D
P	235,936,697	4,238	1,024,457,557,943	2,075,188,958	D
P+ED+TH	236,145,176	212,716	1,024,492,532,320	2,110,163,335	D
P+TH	236,062,803	130,344	1,024,501,348,645	2,118,979,660	D
CM+P+ED+TH	236,275,967	343,508	1,026,127,481,768	3,745,112,783	Ex.D
CM+P+TH	236,204,714	272,254	1,026,143,374,523	3,761,005,538	D
CM+P+ED	236,196,230	263,770	1,026,144,701,581	3,762,332,596	D
CM+P	236,117,644	185,185	1,026,154,365,196	3,771,996,211	D
HS+ED	236,104,850	172,391	1,027,491,836,190	5,109,467,205	D
HS	236,008,921	76,461	1,027,492,009,483	5,109,640,498	D
HS+ED+TH	236,253,752	321,292	1,027,540,906,227	5,158,537,242	D
HS+TH	236,160,985	228,526	1,027,545,554,258	5,163,185,273	D
CM+HS+ED	236,326,072	393,612	1,029,207,007,189	6,824,638,204	Ex.D
CM+HS+ED+TH	236,449,504	517,045	1,029,210,615,443	6,828,246,458	29,514
CM+HS	236,235,107	302,647	1,029,211,383,949	6,829,014,964	D
CM+HS+TH	236,364,392	431,933	1,029,220,325,882	6,837,956,897	D
P+HS	236,013,964	81,505	1,029,571,604,283	7,189,235,298	D
P+HS+ED	236,109,514	177,055	1,029,575,006,982	7,192,637,997	D
P+HS+ED+TH	236,258,317	325,857	1,029,635,201,675	7,252,832,690	D
P+HS+TH	236,165,576	233,116	1,029,636,609,315	7,254,240,330	D
CM+P+HS+ED	236,330,136	397,676	1,031,307,462,643	8,925,093,658	D
CM+P+HS	236,239,448	306,988	1,031,308,497,584	8,926,128,599	D
CM+P+HS+ED+TH	236,453,017	520,557	1,031,320,804,786	8,938,435,801	600,737
CM+P+HS+TH	236,368,316	435,857	1,031,327,616,052	8,945,247,067	D

*ND=Not dominated; Ex.D=Dominated by extension; D=Dominated; Strategies that are dominated are more costly and less effective than not dominated strategies. Strategies that are dominated by extension are more costly and less effective than the linear scale-up not dominated strategies. SQ=Status Quo; CM=Contingency Management; P=Psychotherapy; HS=Hub and Spoke; ED=Emergency Department Initiation; TH=Telehealth

© 2023 Claypool AL et al. *JAMA Health Forum.*

eTable 11: Expected overdose deaths for each strategy, with varying coverage interventions

	**Overdose Deaths Averted Compared to SQ**		
**Strategy***	**5% Coverage**	**10% Coverage***	**20% Coverage**
SQ	0	0	0
CM	1,870	3,530	6,230
P	40	80	160
HS	520	940	1,560
ED	560	1,110	2,180
TH	1,250	2,420	4,460
CM+P	1,910	3,590	6,320
CM+HS	2,530	5,050	10,010
P+HS	560	1,030	1,750
CM+P+HS	2,570	5,130	10,170
CM+ED	2,400	4,500	7,840
HS+ED	1,100	2,130	4,000
P+ED	600	1,190	2,340
CM+P+ED	2,440	4,560	7,930
CM+HS+ED	3,100	6,170	12,260
P+HS+ED	1,140	2,210	4,190
CM+P+HS+ED	3,140	6,250	12,410
CM+TH	2,990	5,390	8,690
P+TH	1,290	2,490	4,580
HS+TH	1,860	3,690	7,280
ED+TH	1,790	3,440	6,270
CM+P+TH	3,020	5,440	8,750
CM+HS+TH	3,780	7,460	14,620
P+HS+TH	1,900	3,780	7,450
CM+ED+TH	3,490	6,260	9,950
HS+ED+TH	2,430	4,840	9,610
P+ED+TH	1,830	3,510	6,380
CM+P+ED+TH	3,520	6,320	10,000
CM+HS+ED+TH	4,320	8,500	16,510
P+HS+ED+TH	2,470	4,920	9,780
CM+P+HS+TH	3,820	7,530	14,740
All interventions	4,360	8,570	16,620

SQ=Status Quo; CM=Contingency Management; P=Psychotherapy; HS=Hub and Spoke; ED=Emergency Department Initiation; TH=Telehealth

© 2023 Claypool AL et al. *JAMA Health Forum.*

eTable 12: Expected intervention costs, QALYs, and cost per QALY gained for each strategy; 5% coverage of all interventions

Strategy*	Total QALYs	QALYs Gained Compared to SQ	Total Cost (2021 USD)	Incremental Cost Compared to SQ (2021 USD)	ICER** (2021 USD per QALY gained)
CM+ED+TH	236,128,545	196,086	1,569,854,672,955	-1,633,812,509	ND
CM+TH	236,087,750	155,290	1,570,217,085,884	-1,271,399,581	D
ED+TH	236,042,601	110,141	1,570,220,252,279	-1,268,233,186	D
TH	235,998,853	66,394	1,570,613,810,063	-874,675,402	D
CM+ED	236,073,910	141,450	1,570,614,390,413	-874,095,051	D
CM+P+ED+TH	236,129,931	197,471	1,570,877,812,413	-610,673,052	Ex.D
CM	236,031,265	98,806	1,570,999,301,059	-489,184,406	D
ED	235,977,620	45,161	1,571,081,622,935	-406,862,530	D
P+ED+TH	236,044,384	111,925	1,571,233,171,153	-255,314,312	D
CM+P+TH	236,089,522	157,062	1,571,239,740,418	-248,745,047	D
SQ	235,932,459	0	1,571,488,485,465	0	D
P+TH	236,000,822	68,362	1,571,625,803,511	137,318,046	D
CM+P+ED	236,075,945	143,486	1,571,628,821,067	140,335,603	D
CM+HS+ED+TH	236,194,741	262,282	1,571,693,318,212	204,832,747	27,776
CM+P	236,033,139	100,680	1,572,012,547,399	524,061,934	D
CM+HS+TH	236,150,589	218,129	1,572,087,990,541	599,505,076	D
P+ED	235,979,629	47,170	1,572,091,228,311	602,742,846	D
HS+ED+TH	236,093,307	160,848	1,572,204,080,747	715,595,282	D
P	235,934,493	2,033	1,572,497,078,734	1,008,593,270	D
CM+HS+ED	236,129,293	196,833	1,572,554,958,131	1,066,472,666	D
HS+TH	236,047,411	114,951	1,572,618,452,932	1,129,967,467	D
All interventions	236,196,651	264,192	1,572,711,526,376	1,223,040,911	533,126
CM+HS	236,083,936	151,477	1,572,968,225,503	1,479,740,039	D
CM+P+HS+TH	236,152,662	220,202	1,573,104,987,186	1,616,501,721	D
HS+ED	236,021,211	88,751	1,573,133,993,087	1,645,507,622	D
P+HS+ED+TH	236,095,644	163,185	1,573,217,472,184	1,728,986,719	D
HS	235,974,526	42,066	1,573,554,435,505	2,065,950,040	D
CM+P+HS+ED	236,131,301	198,842	1,573,570,360,353	2,081,874,888	D
P+HS+TH	236,049,621	117,162	1,573,631,025,479	2,142,540,014	D
CM+P+HS	236,086,127	153,667	1,573,982,772,379	2,494,286,914	D
P+HS+ED	236,023,461	91,002	1,574,144,342,820	2,655,857,355	D
P+HS	235,976,838	44,379	1,574,564,312,021	3,075,826,556	D

*ND=Not dominated; Ex.D=Dominated by extension; D=Dominated; Strategies that are dominated are more costly and less effective than not dominated strategies. Strategies that are dominated by extension are more costly and less effective than the linear scale-up not dominated strategies. SQ=Status Quo; CM=Contingency Management; P=Psychotherapy; HS=Hub and Spoke; ED=Emergency Department Initiation; TH=Telehealth

© 2023 Claypool AL et al. *JAMA Health Forum.*

eTable 13: Expected intervention costs, QALYs, and cost per QALY gained for each strategy; 20% coverage of all interventions

Strategy*	Total QALYs	QALYs Gained Compared to SQ	Total Cost (2021 USD)	Incremental Cost Compared to SQ (2021 USD)	ICER** (2021 USD per QALY gained)
ED+TH	236,307,321	374,862	1,567,026,839,182	-4,461,646,283	ND
CM+ED+TH	236,431,297	498,837	1,567,624,643,977	-3,863,841,488	4,822
TH	236,159,469	227,009	1,568,356,442,768	-3,132,042,697	D
CM+TH	236,326,069	393,609	1,568,575,585,595	-2,912,899,870	D
CM+ED	236,371,400	438,941	1,569,220,083,264	-2,268,402,201	D
ED	236,109,274	176,815	1,569,901,069,570	-1,587,415,895	D
CM	236,238,794	306,334	1,570,398,972,352	-1,089,513,113	D
P+ED+TH	236,311,909	379,450	1,571,150,190,908	-338,294,557	D
SQ	235,932,459	0	1,571,488,485,465	0	D
CM+P+ED+TH	236,431,835	499,376	1,571,819,421,686	330,936,221	Ex.D
P+TH	236,164,866	232,407	1,572,461,890,461	973,404,996	D
CM+P+TH	236,327,581	395,122	1,572,758,179,780	1,269,694,315	D
CM+P+ED	236,374,469	442,010	1,573,372,147,833	1,883,662,368	D
CM+HS+ED+TH	236,941,461	1,009,002	1,573,507,503,982	2,019,018,517	11,531
P+ED	236,117,297	184,837	1,573,951,938,634	2,463,453,169	D
CM+P	236,242,631	310,171	1,574,541,542,756	3,053,057,291	D
CM+HS+TH	236,786,247	853,788	1,574,909,646,168	3,421,160,703	D
HS+ED+TH	236,572,427	639,968	1,575,194,961,321	3,706,475,856	D
P	235,940,957	8,497	1,575,524,530,836	4,036,045,371	D
CM+HS+ED	236,718,648	786,189	1,576,632,422,842	5,143,937,377	D
HS+TH	236,383,451	450,991	1,576,897,067,370	5,408,581,905	D
CM+P+HS+ED+TH	236,946,595	1,014,135	1,577,713,054,070	6,224,568,605	819,183
CM+HS	236,536,041	603,581	1,578,274,709,799	6,786,224,334	D
CM+P+HS+TH	236,792,952	860,492	1,579,096,768,934	7,608,283,469	D
HS+ED	236,257,514	325,055	1,579,176,938,229	7,688,452,764	D
P+HS+ED+TH	236,581,847	649,388	1,579,301,634,077	7,813,148,612	D
CM+P+HS+ED	236,727,213	794,754	1,580,775,416,028	9,286,930,563	D
HS	236,060,309	127,850	1,580,953,895,363	9,465,409,898	D
P+HS+TH	236,393,207	460,748	1,580,988,020,309	9,499,534,844	D
CM+P+HS	236,544,919	612,460	1,582,397,278,241	10,908,792,776	D
P+HS+ED	236,267,909	335,450	1,583,219,048,683	11,730,563,218	D
P+HS	236,071,128	138,669	1,584,980,289,212	13,491,803,747	D

*ND=Not dominated; Ex.D=Dominated by extension; D=Dominated; Strategies that are dominated are more costly and less effective than not dominated strategies. Strategies that are dominated by extension are more costly and less effective than the linear scale-up not dominated strategies. SQ=Status Quo; CM=Contingency Management; P=Psychotherapy; HS=Hub and Spoke; ED=Emergency Department Initiation; TH=Telehealth

© 2023 Claypool AL et al. *JAMA Health Forum.*

eTable 14: Quality-adjusted life years for each health state in SOURCE

Health Stock	QALY Value	QALY Range	Source
Non-disordered heroin use	0.574	(0.538-0.611)	(70)
Rx misuse no heroin use in the past year	0.694	(0.660,0.727)	(70)
Rx OUD no heroin use in the past year no MOUD	0.626	(0.591-0.661)	(70)
Rx OUD with heroin use in the past year no MOUD	0.569	-	Calculated
HUD no MOUD	0.512	(0.475-0.549)	(70)
Rx OUD no heroin in buprenorphine treatment	0.766	(0.7395-0.7925)	(70)
Rx OUD no heroin in methadone treatment	0.766	(0.7395-0.7925)	(70)
Rx OUD no heroin in Vivitrol treatment	0.766	(0.7395-0.7925)	(70)
Rx OUD with heroin in buprenorphine treatment	0.766	(0.7395-0.7925)	(70)
Rx OUD with heroin in methadone treatment	0.766	(0.7395-0.7925)	(70)
Rx OUD with heroin in Vivitrol treatment	0.766	(0.7395-0.7925)	(70)
HUD in buprenorphine treatment	0.766	(0.7395-0.7925)	(70)
HUD in methadone treatment	0.766	(0.7395-0.7925)	(70)
HUD in Vivitrol treatment	0.766	(0.7395-0.7925)	(70)
Rx OUD no heroin in remission	0.807	(0.78-0.834)	(70)
Rx OUD no heroin in stable remission	0.807	(0.78-0.834)	(70)
Rx OUD with heroin in remission	0.807	(0.78-0.834)	(70)
Rx OUD with heroin in stable remission	0.807	(0.78-0.834)	(70)
HUD in remission	0.807	(0.78-0.834)	(70)
HUD in stable remission	0.807	(0.78-0.834)	(70)

MOUD=Medication for opioid use disorder; OUD=Opioid use disorder; Rx OUD=Opioid use disorder involving prescription opioids; HUD=Heroin-use disorder; Opioid use disorder involving heroin

References:

1. Lim TY, Stringfellow EJ, Stafford CA, DiGennaro C, Homer JB, Wakeland W, et al. Modeling the evolution of the US opioid crisis for national policy development. Proceedings of the National Academy of Sciences [Internet]. 2022 Jun 7 [cited 2022 Jun 5];119(23). Available from: https://pnas.org/doi/full/10.1073/pnas.2115714119
2. Stringfellow EJ, Lim TYL, Humphreys K, DiGennaro C, Stafford C, Beaulieu E, et al. Reducing opioid use disorder and overdose deaths in the United States: A dynamic modeling analysis. Sci Adv [Internet]. 2022 [cited 2022 Jun 27];in press:8147. Available from: https://www.science.org/doi/10.1126/sciadv.abm8147
3. S. Aronowitz, C. Behrends, M. Lowenstein, B. Schackman JW. Lowering the Barriers to Medication Treatment for People with Opioid Use Disorder - CHERISH [Internet]. 2022 [cited 2022 Jul 5]. Available from: https://cherishresearch.org/2022/01/lowering-the-barriers-to-medication-treatment-for-people-with-opioid-use-disorder-2/
4. Ouzzani M, Hammady H, Fedorowicz Z, Elmagarmid A. Rayyan- a web and mobile app for systematic reviews. Syst Rev. 2016;5(210).
5. Fairley M, Humphreys K, Joyce VR, Bounthavong M, Trafton J, Combs A, et al. Cost-effectiveness of treatments for opioid use disorder. JAMA Psychiatry [Internet]. 2021 Mar 31 [cited 2021 Mar 30];4026:1–11. Available from: https://jamanetwork.com/journals/jamapsychiatry/fullarticle/2778020
6. Brooklyn JR, Sigmon SC. Vermont Hub-and-Spoke Model of Care for Opioid Use Disorder: Development, Implementation, and Impact. J Addict Med. 2017;11(4).
7. D'Onofrio G, O'Connor PG, Pantalon M V., Chawarski MC, Busch SH, Owens PH, et al. Emergency department-initiated buprenorphine/naloxone treatment for opioid dependence: a randomized clinical trial. JAMA. 2015 Apr 28;313(16):1636–44.
8. Jennings LK, Lane S, McCauley J, Moreland A, Hartwell K, Haynes L, et al. Retention in Treatment after Emergency Department-Initiated Buprenorphine. J Emerg Med. 2021 Sep 1;61(3):211–21.
9. Kaucher KA, Caruso EH, Sungar G, Gawenus L, Hurlbut K, Colon Sanchez D, et al. Evaluation of an emergency department buprenorphine induction and medication-assisted treatment referral program. Am J Emerg Med. 2019;38(2):300–4.
10. Bogan C, Jennings L, Haynes L, Barth K, Moreland A, Oros M, et al. Implementation of emergency department-initiated buprenorphine for opioid use disorder in a rural southern state. J Subst Abuse Treat. 2020 Mar 1;112S:73–8.
11. Lin LA, Fortney JC, Bohnert ASB, Coughlin LN, Zhang L, Piette JD. Comparing telemedicine to in-person buprenorphine treatment in U.S. veterans with opioid use disorder. J Subst Abuse Treat. 2022;133.
12. Reif S, Brolin MF, Stewart MT, Fuchs TJ, Speaker E, Mazel SB. The Washington State Hub and Spoke Model to increase access to medication treatment for opioid use disorders. J Subst Abuse Treat. 2020;108:33–9.
13. Busch SH, Fiellin DA, Chawarski MC, Owens PH, Pantalon M V., Hawk K, et al. Cost-effectiveness of emergency department-initiated treatment for opioid dependence. Addiction (Abingdon, England). 2017 Nov 1;112(11):2002–10.
14. Linas BP, Savinkina A, Madushani RWMA, Wang J, Eftekhari Yazdi G, Chatterjee A, et al. Projected estimates of opioid mortality after community-level interventions. JAMA Netw Open. 2021 Feb 15;4(2):e2037259.

© 2023 Claypool AL et al. *JAMA Health Forum.*

15. Richter KP, Shireman TI, Ellerbeck EF, Cupertino AP, Cox LS, Preacher KJ, et al. Comparative and Cost Effectiveness of Telemedicine Versus Telephone Counseling for Smoking Cessation. J Med Internet Res 2015;17(5):e113 https://www.jmir.org/2015/5/e113 [Internet]. 2015 May 8 [cited 2022 Jul 5];17(5):e3975. Available from: https://www.jmir.org/2015/5/e113
16. Sullivan PW, Ghushchyan V. Preference-Based EQ-5D index scores for chronic conditions in the United States. Med Decis Making. 2006;26(4):410–20.
17. Murphy SM. The cost of opioid use disorder and the value of aversion. Drug Alcohol Depend. 2020 Dec 1;217.
18. Mitchell EM. Concentration of Healthcare Expenditures and Selected Characteristics of High Spenders, US Civilian Noninstitutionalized Population, 2018 [Internet]. 2021. Available from: https://meps.ahrq.gov/data_files/publications/st533/stat533.pdf
19. Baser O, Xie L, Mardekian J, Schaaf D, Wang L, Joshi A V. Prevalence of diagnosed opioid abuse and its economic burden in the veterans health administration. Pain Practice [Internet]. 2014 Jun 1 [cited 2021 Jun 16];14(5):437–45. Available from: https://onlinelibrary.wiley.com/doi/full/10.1111/papr.12097
20. Kirson NY, Shei A, Rice JB, Enloe CJ, Bodnar K, Birnbaum HG, et al. The burden of undiagnosed opioid abuse among the commercially insured. Pain Medicine (United States) [Internet]. 2015 Jul [cited 2021 Jun 25];16(7):1325–32. Available from: https://academic.oup.com/painmedicine/article-lookup/doi/10.1111/pme.12768
21. Behrends CN, Paone D, Nolan ML, Tuazon E, Murphy SM, Kapadia SN, et al. Estimated impact of supervised injection facilities on overdose fatalities and healthcare costs in New York City. J Subst Abuse Treat [Internet]. 2019 Nov 1 [cited 2021 Jun 27];106:79–88. Available from: https://pubmed.ncbi.nlm.nih.gov/31540615/
22. Premier Inc. Opioid overdoses costing U.S. hospitals an estimated $11 billion annually [Internet]. [cited 2021 Jun 27]. Available from: https://www.premierinc.com/newsroom/press-releases/opioid-overdoses-costing-u-s-hospitals-an-estimated-11-billion-annually
23. Coffin PO, Sullivan SD. Cost-effectiveness of distributing naloxone to heroin users for lay overdose reversal. Ann Intern Med [Internet]. 2013 Jan 1 [cited 2021 Jun 27];158(1):1–9. Available from: https://pubmed.ncbi.nlm.nih.gov/23277895/
24. Jiang Y, McDonald J V., Koziol J, McCormick M, Viner-Brown S, Alexander-Scott N. Can emergency department, hospital discharge, and death data be used to monitor burden of drug overdose in Rhode Island? Journal of Public Health Management and Practice [Internet]. 2017 [cited 2021 Jun 27];23(5):499–506. Available from: https://pubmed.ncbi.nlm.nih.gov/28009694/
25. Murphy SM, McCollister KE, Leff JA, Yang X, Jeng PJ, Lee JD, et al. Cost-effectiveness of buprenorphine–naloxone versus extended-release naltrexone to prevent opioid relapse. Ann Intern Med. 2019 Jan 15;170(2):90–8.
26. Neumann PJ, Sanders GD, Russell LB, Siegel JE, Ganiats TG. Cost-Effectiveness in Health and Medicine. Second Edi. New York: Oxford University Press; 2017.
27. Arias E, Xu J. United States Life Tables, 2018. 2020;69(12). Available from: https://www.cdc.gov/nchs/data/nvsr/nvsr69/nvsr69-12-508.pdf
28. United States Census Bureau. Current Population Survey 2020 Annual Social and Economic (ASEC) Supplement [Internet]. 2020. Available from: https://www2.census.gov/programs-surveys/cps/techdocs/cpsmar20.pdf
29. United States Bureau of Labor Statistics. Consumer Expenditure Survey,2019. 2020.

© 2023 Claypool AL et al. *JAMA Health Forum.*

30. Sanders GD, Neumann PJ, Basu A, Brock DW, Feeny D, Krahn M, et al. Recommendations for Conduct, Methodological Practices, and Reporting of Cost-effectiveness Analyses: Second Panel on Cost-Effectiveness in Health and Medicine. JAMA [Internet]. 2016 Sep 13 [cited 2023 Mar 5];316(10):1093–103. Available from: https://pubmed.ncbi.nlm.nih.gov/27623463/
31. Ling W, Hillhouse M, Ang A, Jenkins J, Fahey J. Comparison of behavioral treatment conditions in buprenorphine maintenance. Addiction [Internet]. 2013 Oct 1 [cited 2021 Jul 11];108(10):1788–98. Available from: https://onlinelibrary.wiley.com/doi/full/10.1111/add.12266
32. Fiellin DA, Pantalon M V., Chawarski MC, Moore BA, Sullivan LE, O'Connor PG, et al. Counseling plus Buprenorphine–Naloxone Maintenance Therapy for Opioid Dependence. New England Journal of Medicine. 2006;355(4).
33. Polsky D, Glick HA, Yang J, Subramaniam GA, Poole SA, Woody GE. Cost-effectiveness of extended buprenorphine-naloxone treatment for opioid-dependent youth: Data from a randomized trial. Addiction. 2010;105(9).
34. Tacke U, Uosukainen H, Kananen M, Kontra K, Pentikäinen H. A pilot study about the feasibility and cost-effectiveness of electronic compliance monitoring in substitution treatment with buprenorphine-naloxone combination. J Opioid Manag. 2009;5(6).
35. Winstanley EL, Lander LR, Berry JH, Mahoney JJ, Zheng W, Herschler J, et al. West Virginia's model of buprenorphine expansion: Preliminary results. J Subst Abuse Treat. 2020;108.
36. Levin FR, Bisaga A, Sullivan MA, Williams AR, Cates-Wessel K. A review of a national training initiative to increase provider use of MAT to address the opioid epidemic. Vol. 25, American Journal on Addictions. 2016.
37. Komaromy M, Duhigg D, Metcalf A, Carlson C, Kalishman S, Hayes L, et al. Project ECHO (Extension for Community Healthcare Outcomes): A new model for educating primary care providers about treatment of substance use disorders. Subst Abus [Internet]. 2016 Jan 2 [cited 2022 Jan 16];37(1):20–4. Available from: https://pubmed.ncbi.nlm.nih.gov/26848803/
38. Auty SG, Stein MD, Walley AY, Drainoni ML. Buprenorphine waiver uptake among nurse practitioners and physician assistants: The role of existing waivered prescriber supply. J Subst Abuse Treat [Internet]. 2020 Aug;115:108032. Available from: https://linkinghub.elsevier.com/retrieve/pii/S0740547219305537
39. Tong ST, Hochheimer CJ, Peterson LE, Krist AH. Buprenorphine provision by early career family physicians. Ann Fam Med [Internet]. 2018 Sep 1 [cited 2021 Jun 22];16(5):443–6. Available from: https://www.annfammed.org/content/16/5/443
40. Wen H, Hockenberry JM, Pollack HA. Association of Buprenorphine-Waivered Physician Supply With Buprenorphine Treatment Use and Prescription Opioid Use in Medicaid Enrollees. JAMA Netw Open [Internet]. 2018 Sep 7 [cited 2021 Jun 22];1(5):e182943. Available from: https://jamanetwork.com/
41. Foster SD, Lee K, Edwards C, Pelullo AP, Khatri UG, Lowenstein M, et al. Providing Incentive for Emergency Physician X-Waiver Training: An Evaluation of Program Success and Postintervention Buprenorphine Prescribing. Ann Emerg Med. 2020 Aug 1;76(2):206–14.
42. Stokes DC, Perrone J. Increasing Short- and Long-Term Buprenorphine Treatment Capacity. Academic Medicine. 2021;Online Ahe.
43. Lin LA, Fortney JC, Bohnert ASB, Coughlin LN, Zhang L, Piette JD. Comparing telemedicine to in-person buprenorphine treatment in U.S. veterans with opioid use disorder. J Subst Abuse Treat. 2022 Feb 1;133:108492.

© 2023 Claypool AL et al. *JAMA Health Forum.*

44. Weintraub E, Seneviratne C, Anane J, Coble K, Magidson J, Kattakuzhy S, et al. Mobile Telemedicine for Buprenorphine Treatment in Rural Populations with Opioid Use Disorder. JAMA Netw Open. 2021;4(8).
45. Belcher AM, Coble K, Cole TO, Welsh CJ, Whitney A, Weintraub E. Buprenorphine Induction in a Rural Maryland Detention Center During COVID-19: Implementation and Preliminary Outcomes of a Novel Telemedicine Treatment Program for Incarcerated Individuals With Opioid Use Disorder. Front Psychiatry. 2021;12.
46. Weintraub E, Greenblatt AD, Chang J, Himelhoch S, Welsh C. Expanding access to buprenorphine treatment in rural areas with the use of telemedicine. American Journal on Addictions. 2018;27(8).
47. Harris R, Rosecrans A, Zoltick M, Willman C, Saxton R, Cotterell M, et al. Utilizing telemedicine during COVID-19 pandemic for a low-threshold, street-based buprenorphine program. Drug Alcohol Depend. 2022;230.
48. Tofighi B, McNeely J, Walzer D, Fansiwala K, Demner A, Chaudhury CS, et al. A Telemedicine Buprenorphine Clinic to Serve New York City: Initial Evaluation of the NYC Public Hospital System's Initiative to Expand Treatment Access during the COVID-19 Pandemic. J Addict Med. 2022;16(1).
49. Rahman F, Evans NK, Bernhardt J. Access to OUD Treatment and Maintenance of Sobriety amid the COVID-19 Pandemic. Subst Use Misuse. 2021;56(7).
50. Yeo EJ, Kralles H, Sternberg D, McCullough D, Nadanasabesan A, Mayo R, et al. Implementing a low-threshold audio-only telehealth model for medication-assisted treatment of opioid use disorder at a community-based non-profit organization in Washington, D.C. Harm Reduct J. 2021;18(1).
51. Ruetsch C, Tkacz J, McPherson TL, Cacciola J. The effect of telephonic patient support on treatment for opioid dependence: Outcomes at one year follow-up. Addictive Behaviors. 2012;37(5).
52. Sahu N, Chen PH, Shimoni N. Telehealth to improve continuity for patients receiving buprenorphine treatment for opioid use disorder. Ann Fam Med [Internet]. 2022 Apr 1 [cited 2023 Feb 19];20(20 Suppl 1). Available from: https://pubmed.ncbi.nlm.nih.gov/35947491/
53. Vakkalanka JP, Lund BC, Ward MM, Arndt S, Field RW, Charlton M, et al. Telehealth Utilization Is Associated with Lower Risk of Discontinuation of Buprenorphine: a Retrospective Cohort Study of US Veterans. J Gen Intern Med [Internet]. 2022 May 1 [cited 2023 Feb 19];37(7):1610–8. Available from: https://pubmed.ncbi.nlm.nih.gov/34159547/
54. Samuels EA, Khatri UG, Snyder H, Wightman RS, Tofighi B, Krawczyk N. Buprenorphine Telehealth Treatment Initiation and Follow-Up During COVID-19. J Gen Intern Med [Internet]. 2022 Apr 1 [cited 2023 Feb 19];37(5):1331–3. Available from: https://pubmed.ncbi.nlm.nih.gov/34981357/
55. Lin LA, Zhang L, Kim HM, Frost MC. Impact of COVID-19 Telehealth Policy Changes on Buprenorphine Treatment for Opioid Use Disorder. Am J Psychiatry [Internet]. 2022 Oct 1 [cited 2023 Feb 19];179(10):740–7. Available from: https://pubmed.ncbi.nlm.nih.gov/35899380/
56. Kaur J, Mania I, Tirupathi R, Polavarapu L. Impact of telemedicine on retention in Medications for Opioid Use Disorder (MOUD) treatment with buprenorphine in the times of COVID-19 pandemic: A retrospective chart review. Journal of Rural Mental Health. 2022 Apr;46(2):75–81.
57. Frost MC, Zhang L, Kim HM, Lin L. Use of and Retention on Video, Telephone, and In-Person Buprenorphine Treatment for Opioid Use Disorder During the COVID-19 Pandemic. JAMA Netw

© 2023 Claypool AL et al. *JAMA Health Forum.*

Open [Internet]. 2022 Oct 12 [cited 2023 Feb 19];5(10):E2236298. Available from: https://pubmed.ncbi.nlm.nih.gov/36223118/
58. Xu CQ, Smith AC, Scuffham PA, Wootton R. A cost minimisation analysis of a telepaediatric otolaryngology service. BMC Health Serv Res. 2008;8.
59. Armstrong AW, Dorer DJ, Lugn NE, Kvedar JC. Economic evaluation of interactive teledermatology compared with conventional care. Telemedicine Journal and e-Health. 2007;13(2).
60. Paré G, Sicotte C, St.-Jules D, Gauthier R. Cost-minimization analysis of a telehomecare program for patients with chronic obstructive pulmonary disease. Telemedicine Journal and e-Health. 2006;12(2).
61. Labiris G, Tsitlakidis C, Niakas D. Retrospective economic evaluation of the Hellenic Air Force teleconsultation project. J Med Syst. 2005;29(5).
62. Scuffham PA, Steed M. An Economic Evaluation of the Highlands and Islands Teledentistry Project. J Telemed Telecare. 2002;8(3).
63. Bergmo TS. A cost-minimization analysis of a realtime teledermatology service in northern Norway. J Telemed Telecare. 2000;6(5).
64. McCue MJ, Mazmanian PE, Hampton C, Marks TK, Fisher E, Parpart F, et al. The case of Powhatan Correctional Center/Virginia Department of Corrections and Virginia Commonwealth University/Medical College of Virginia. Telemedicine Journal. 1997;3(1).
65. Frederix I, Hansen D, Coninx K, Vandervoort P, Vandijck D, Hens N, et al. Effect of comprehensive cardiac telerehabilitation on one-year cardiovascular rehospitalization rate, medical costs and quality of life: A cost-effectiveness analysis. Eur J Prev Cardiol. 2016;23(7).
66. Greving JP, Kaasjager HAH, Vernooij JWP, Hovens MMC, Wierdsma J, Grandjean HMH, et al. Cost-effectiveness of a nurse-led internet-based vascular risk factor management programme: Economic evaluation alongside a randomised controlled clinical trial. BMJ Open. 2015;5(5).
67. Nguyen H V., Tan GSW, Tapp RJ, Mital S, Ting DSW, Wong HT, et al. Cost-effectiveness of a National Telemedicine Diabetic Retinopathy Screening Program in Singapore. Ophthalmology. 2016;123(12).
68. Thomas S, Hodge W, Malvankar-Mehta M. The cost-effectiveness analysis of teleglaucoma screening device. PLoS One. 2015;10(9).
69. Zanaboni P, Landolina M, Marzegalli M, Lunati M, Perego GB, Guenzati G, et al. Cost-utility analysis of the EVOLVO study on remote monitoring for heart failure patients with implantable defibrillators: Randomized controlled trial. J Med Internet Res. 2013;15(5).
70. Wittenberg E, Bray JW, Aden B, Gebremariam A, Nosyk B, Schackman BR. Measuring benefits of opioid misuse treatment for economic evaluation: Health-related quality of life of opioid-dependent individuals and their spouses as assessed by a sample of the US population. Addiction. 2016 Apr 1;111(4):675–84.

© 2023 Claypool AL et al. *JAMA Health Forum.*
