# Supplementary material for: Cost-effectiveness of Increasing Buprenorphine Treatment Initiation, Duration, and Capacity Among Individuals Who Use Opioids
Source: JAMA Health Forum. 2023 May 19;4(5):e231080. doi: 10.1001/jamahealthforum.2023.1080 (PMC10199347; doi:10.1001/jamahealthforum.2023.1080)
Supplement: Supplement 2. — Data sharing statement [file jamahealthforum-e231080-s002.pdf]

Data Sharing Statement

Claypool. Cost-Effectiveness of Increasing Buprenorphine Treatment Initiation, Duration, and Capacity Among Individuals Who Use Opioids. *JAMA Health Forum*. Published May 19, 2023. doi:10.1001/jamahealthforum.2023.1080

Data
Data available: Yes
Data types: Data (not involving human participants)
How to access data: All data, code, and materials are available in the Supplementary Materials and the online repository: github.com/annclay/CEA_Buprenorphine_Expansion_US
When available: With publication

Supporting Documents
Document types: None

Additional Information
Who can access the data: Everyone
Types of analyses: For any purpose
Mechanisms of data availability: All data are shared publicly
Any additional restrictions: No restrictions
